# Lignolytic-consortium omics analyses reveal novel genomes and pathways involved in lignin modification and valorization

**DOI:** 10.1186/s13068-018-1073-4

**Published:** 2018-03-22

**Authors:** Eduardo C. Moraes, Thabata M. Alvarez, Gabriela F. Persinoti, Geizecler Tomazetto, Livia B. Brenelli, Douglas A. A. Paixão, Gabriela C. Ematsu, Juliana A. Aricetti, Camila Caldana, Neil Dixon, Timothy D. H. Bugg, Fabio M. Squina

**Affiliations:** 1 Laboratório Nacional de Ciência e Tecnologia do Bioetanol, Centro Nacional de Pesquisa em Energia e Materiais, Campinas, Brazil; 2 Master Program in Industrial Biotechnology, Universidade Positivo (UP), Curitiba, Brazil; 3 Manchester Institute of Biotechnology, School of Chemistry, University of Manchester, Manchester, UK; 4 Department of Chemistry, University of Warwick, Coventry, UK; 5 Programa de Processos Tecnológicos e Ambientais, Universidade de Sorocaba, Sorocaba, Brazil

**Keywords:** Lignin, Aromatic compound degradation, Metagenome, Vanillin, Ferulic acid

## Abstract

**Background:**

Lignin is a heterogeneous polymer representing a renewable source of aromatic and phenolic bio-derived products for the chemical industry. However, the inherent structural complexity and recalcitrance of lignin makes its conversion into valuable chemicals a challenge. Natural microbial communities produce biocatalysts derived from a large number of microorganisms, including those considered unculturable, which operate synergistically to perform a variety of bioconversion processes. Thus, metagenomic approaches are a powerful tool to reveal novel optimized metabolic pathways for lignin conversion and valorization.

**Results:**

The lignin-degrading consortium (LigMet) was obtained from a sugarcane plantation soil sample. The LigMet taxonomical analyses (based on 16S rRNA) indicated prevalence of *Proteobacteria*, *Actinobacteria* and *Firmicutes* members, including the *Alcaligenaceae* and *Micrococcaceae* families, which were enriched in the LigMet compared to sugarcane soil. Analysis of global DNA sequencing revealed around 240,000 gene models, and 65 draft bacterial genomes were predicted. Along with depicting several peroxidases, dye-decolorizing peroxidases, laccases, carbohydrate esterases, and lignocellulosic auxiliary (redox) activities, the major pathways related to aromatic degradation were identified, including benzoate (or methylbenzoate) degradation to catechol (or methylcatechol), catechol ortho-cleavage, catechol meta-cleavage, and phthalate degradation. A novel *Paenarthrobacter* strain harboring eight gene clusters related to aromatic degradation was isolated from LigMet and was able to grow on lignin as major carbon source. Furthermore, a recombinant pathway for vanillin production was designed based on novel gene sequences coding for a feruloyl-CoA synthetase and an enoyl-CoA hydratase/aldolase retrieved from the metagenomic data set.

**Conclusion:**

The enrichment protocol described in the present study was successful for a microbial consortium establishment towards the lignin and aromatic metabolism, providing pathways and enzyme sets for synthetic biology engineering approaches. This work represents a pioneering study on lignin conversion and valorization strategies based on metagenomics, revealing several novel lignin conversion enzymes, aromatic-degrading bacterial genomes, and a novel bacterial strain of potential biotechnological interest. The validation of a biosynthetic route for vanillin synthesis confirmed the applicability of the targeted metagenome discovery approach for lignin valorization strategies.

**Electronic supplementary material:**

The online version of this article (10.1186/s13068-018-1073-4) contains supplementary material, which is available to authorized users.

## Background

High global carbon dioxide emission levels and the need for renewable feedstocks for the energy and chemical industries represent great challenges to humankind [1–3]. Lignocellulosic biomass is the most abundant carbon-based material in nature, representing an attractive renewable source to replace oil derivatives and to provide aromatic building blocks for the chemical industry [1–3].

Lignin is one of the major components of plant cell walls, and consists of a highly recalcitrant and heterogeneous polymer network formed via radical coupling reactions involving the three major monolignols: *p*-coumaryl, coniferyl, and sinapyl alcohol, linked by C–C and C–O bonds [4–6]. The rapid expansion of cellulosic biorefineries has increased the production of lignin-rich streams, which, at present, are mainly burned for energy supply [6]. Controlled deconstruction of the macromolecule to produce lower molecular weight compounds is critical in lignin valorization strategies [5, 7]. Nevertheless, examples of chemical depolymerization or acidification treatment of lignin to produce low-molecular-weight aromatic compounds have proven to be methods of interest for adding value to lignin streams [8–10]. The depolymerization of lignin into monomers such as guaiacyl, syringyl, vanillin, and syringaldehyde is of commercial interest due to the potential for application of these molecules in the biofuel, food, cosmetic, and other industrial sectors [11].

There is an increasing interest in the development of biological lignin depolymerization routes using enzymes and microorganisms [12, 13]. In this sense, intensive studies have been performed to elucidate the enzymatic repertoire involved in lignin depolymerization by fungi and bacteria [13, 14]. Lignin degradation by the white-rot fungi has been extensively studied [15]. However, some bacteria, such as *Streptomyces viridosporus* T7A, *Pseudomonas putida* mt-2, *Rhodococcus jostii* RHA1, *Sphingobium* sp. SYK-6, and strains of the genera *Thauera*, *Arthrobacter* and *Rhizobium* have also been described in the process of lignin breakdown [16, 17], as well as possessing metabolic pathways for conversion of this complex substrate into valuable commercial products [11]. For example, *S. paucimobilis* SYK-6 can grow on a variety of lignin-derived compounds and convert them into syringyl or guaiacyl units [18].

Typically, enzyme-based technologies rely on sets of genes produced by a single cultivable organism, whereas natural microbial communities produce biocatalysts derived from a large number of microorganisms, including those considered unculturable, which operate synergistically to perform a variety of bioconversion processes [19, 20]. Alternatively, the development of enriched microbial consortia has been used as a strategy to select microorganisms with genetic content related to a desirable set of biochemical functions [21–25]. Soil is commonly used as a microbial source for enrichment processes due to its high complexity and countless versatility regarding phenotypic traits, offering enormous potential for establishing microbial communities adapted to a variety of environmental factors, such as pH, temperature, pressure, mineral nutrients, and carbon and energy sources [21–24, 26]. The previous studies reported enriched consortia for degradation of lignocellulosic biomass and aromatic compounds, which based on a combination of culture independent methods (e.g., meta-omics approaches) not only disclosed genes and metabolic pathways involved in this degradation process [21, 24, 27], but also elucidated the microbial community structures. Although the enrichment process is a powerful tool for development of a community with the desired functions [21, 24, 27], studies involving lignin and lignin-derived aromatic degradation have been mostly focused on isolation or culture independent methods [14, 17, 18, 28, 29].

**Fig. 1:**
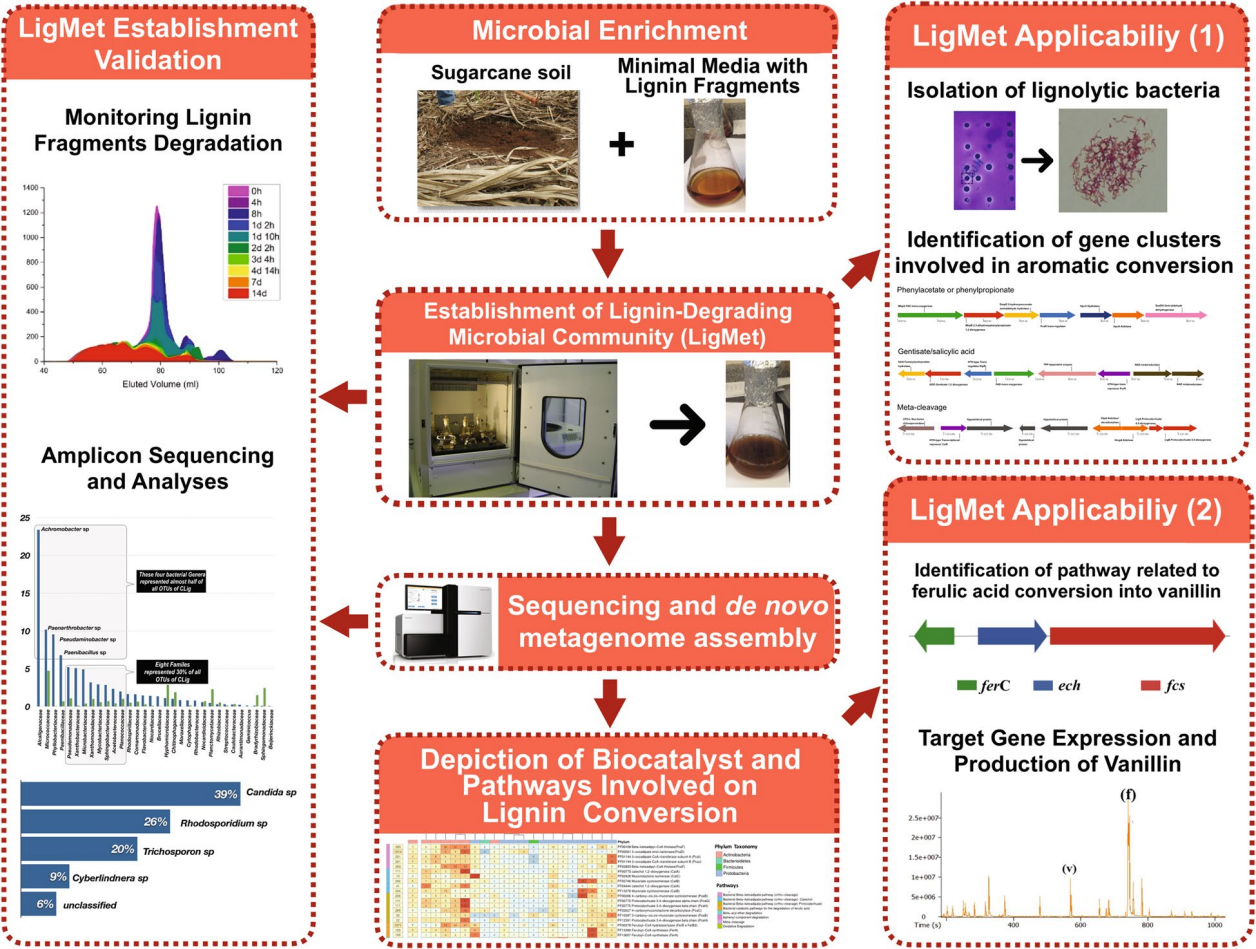
Schematic representation of the targeted metagenomic discovery platform applied in the present study, including establishment and validation of the lignin-degrading consortium, genetic characterization, and potential applications

The present work applied a metagenomic discovery platform, based on targeted community enrichment protocols, to depict bacterial biocatalysts and pathways involved in lignin degradation and aromatic compound conversion (Fig. 1). For this purpose, the lignin-degrading consortium (LigMet) was established using a sugarcane soil sample as inoculum and lignin fragments as the major carbon source in minimal medium. Followed by taxonomical affiliation of microorganism related to lignin degradation in the consortium, metagenomic sequencing allowed for reconstruction of bacterial draft genomes, revealing genes related to lignin depolymerization and metabolic pathways useful in lignin valorization efforts. Moreover, a novel strain *of Paenarthrobacter* capable of metabolizing lignin fragments and containing several aromatic degradation gene clusters in its genome was retrieved from LigMet. Finally, to validate the usefulness of the metagenomic strategy, the biosynthetic route of vanillin production from ferulic acid was applied using novel enzymes retrieved from LigMet corresponding to feruloyl-CoA synthetase (FerA_B3) and enoyl-coA hydratase/aldolase (FerB_B11).

## Methods

### Development of the LigMet

The sugarcane soil sample was collected from a sugarcane farm in Brazil (GPS coordinates: − 22º04′ 77.98, − 48º63′ 52.99′′; Fazenda Tropical). The lignin-degrading consortium (LigMet) was established by adding 1 g soil to a flask containing 100 ml of culture medium supplemented with a stock solution containing soluble lignin (Fig. 1). The stock solution, referred to herein as low-molecular-weight (LW) lignin, consists of a supernatant obtained after the acidification of black liquor generated from the delignification of steam-exploded sugarcane bagasse [30]. The culture media consisted of a 1:1 dilution of the lignin stock solution in distilled water, with the addition of minimum nutrients from Bushnell Haas Broth (Sigma-Aldrich) and pH 7.0. The flask was incubated at 30 °C and 150 rpm. Microbial enrichment was obtained by transferring aliquots (3 ml) of the microbial suspension to fresh medium weekly. This procedure was carried out during 50 weeks prior to the first LigMet analysis. The DNS assay [31] was performed to determine the total reducing sugar consumed. LigMet samples were centrifuged and 100 µl of the supernatant were mixed to 100 µl of the DNS reagent in triplicate as described by the Miller method.

### Lignin degradation evaluation

The consumption of water-soluble lignin fragments present in the stock solution by the microbial community was evaluated by gel permeation chromatography (GPC) in a Superdex^®^ 30 prep grade 127 ml column (GE Healthcare) coupled with an ÄKTA System (GE Healthcare). The GPC gradient was performed isocratically using NaOH 0.1 mol l^−1^ as the eluent at a 0.5 ml min^−1^ flow rate and 20 °C. For each supernatant, 500 µl was diluted in 500 µl of NaOH 0.1 mol l^−1^ prior to injection [8]. The lignin fragments were detected by a UV detector (280 nm) and the molecular weights were determined using different polyphenolic compounds as external standards.

### Sequencing analysis

Total microbial DNA was extracted using the FastDNA Spin Kit for soil (MP Biomedicals) and purified with Power Clean^®^ DNA Clean-Up Kits (Mo Bio Laboratories) according to the manufacturer’s instructions. The hypervariable V4 region of the 16S rRNA and internal transcribed spacer region 2 (ITS2) were amplified using universal primers [32, 33]. PCR products were purified and pooled together in equimolar amounts for sequencing in an Illumina MiSeq system according to the standard procedures. Raw reads were quality assessed using FASTQC (https://www.bioinformatics.babraham.ac.uk/projects/fastqc/), and preprocessed by removing adapters and low-quality sequences, using Trimmomatic [34]. Quality-filtered reads were merged using Pear [35] and further analyzed using the UPARSE pipeline [36]. Finally, the sequences were clustered into operational taxonomic units (OTU) based on a 97% identity using the USEARCH method. Chimeric sequences were identified and removed using the UCHIME method. The representative sequence from each OTU was assigned to taxonomic taxa using Ribosomal Database Project RDP Classifier [37] and UNITE Fungal ITS training sets [38]. For statistical analyses, the Chao1, ACE, Shannon, and Simpson indices, rarefaction curves, and sample coverage (Good’s coverage) were calculated by employing the Phyloseq and Vegan packages using the R statistical software version 3.2.

For shotgun metagenomic sequencing purposes, 50 ng of the total DNA from LigMet were used to prepare an Illumina Nextera library according to the manufacturer’s instructions. The library was validated using an Agilent Bioanalyzer 2100 with the 12000 DNA assay kit (Agilent), quantified by applying the KAPA Biosystem’s next-generation sequencing library qPCR kit (KAPA), and sequenced on Illumina HiSeq 2500 using a 2 × 100 bp paired-end sequencing kit.

Metagenomic reads were quality-filtered using Trimmomatic to remove adaptors and low-quality reads, and then were used for de novo assembly of the metagenome, using IDBA-UD [39]. The assembled contigs were subject to MetaQUAST [40] for quality assessment, and to MetaGeneMark [41] for prokaryotic gene calling, using default parameters. In addition, the contigs were mapped against GenBank NT database, using megablast, and NCBI taxonomic ranks assigned using Blobtools [42], by best sum method. MetaGeneMark was applied, since we were not able to detect eukaryotic sequences in the metagenome assembly. Predicted proteins from LigMet were annotated based on MALT (https://ab.inf.uni-tuebingen.de/software/malt) searches against SwissProt and UniRef90 databases, and were further compared to the Pfam [43], dbCAN [44], and EggNOG databases [45], using the HMMER3 (http://hmmer.janelia.org/) package. A metabolic pathways analysis was performed using the KEGG web application GhostKoala [46].

Metagenomic contigs were submitted to binning by coverage and sequence composition using CONCOCT [47]. Assembled contigs were split in chunks of 10 Kb prior to read mapping using Bowtie2 [48]. Quality of the genome bins was assessed using CheckM [49] to determine genome completeness and contamination, which was based on the presence/absence of sets of colocalized single-copy genes present within a phylogenetic lineage. Bins presenting completeness greater than 75%, contamination less than 10%, and strain heterogeneity less than 25% were selected for further analysis using Phyla-AMPHORA [50], which also incorporates bacterial phylum-level phylogenetic markers for taxonomy assignment.

### Cultivation, isolation, and genomic-based characterization of microorganisms

The LigMet culture broth was serially diluted and plated directly on agar media supplemented with 1:1 (v/v) LW lignin and 0.25% (w/v) high-molecular-weight (HW) insoluble lignin, prepared as described by Rocha et al. [30]. After incubation of the plates at 30 º C for 48 h, 48 selected colonies were transferred again to agar medium supplemented with lignin fractions and incubated at the same condition. The colonies were then covered with a 1.5% (w/v) agar solution supplemented with 0.01% (w/v) Azure-B dye. Colonies showing lignolytic activity by the formation of dye decolorization halos around them were cultivated and their genomic DNA extracted as previously described. Identification was carried out based on the 16S rRNA gene sequencing analysis using BLASTn search against the RDP database with 97% sequence identity and *E*-value of 1e−10. For genome sequencing, the total DNA was sequenced by applying the paired-end and mate-pair library protocols on an Illumina MiSeq platform. Genome reads were first quality-filtered and assembled using Velvet [51], and SSPACE [52] for scaffolding using the mate-pairs reads. Pilon [53] was used to further improve the genome assembly. The assembled genome sequence was imported into the annotation platform Integrated Microbial Genomes (IMG/ER, [54]) for automatic prediction of genes. Finally, the assembled genome of the isolate was compared to the genome bins using Mauve [55] and Mummer (version 3.0 [56],) to determine their similarity.

### Data availability

Raw sequencing reads of the amplicon, metagenome, and genome sequencing analysis have been deposited in the DDBJ/ENA/GenBank under the accession number PRJEB20169. The draft genome sequences of *Paenarthrobacter* sp. HW13 have been deposited in the IMG/ER under Study ID: Gs0118559.

### Cloning and expression of genes involved in vanillin biosynthesis

The biotransformation of ferulic acid into vanillin was based on expression of two genes, *ferA_B3* (MG214406) and *ferB_B11* (MG214407), encoding feruloyl coenzyme A synthetase and enoyl-CoA hydratase, respectively. These genes sequences were obtained from the LigMet metagenomic data set and synthesized by Biomatik (Biomatik Corporation, Canada). The *ferB_B11* gene was inserted into the pET28a-vector, while the *ferA_B3* gene was cloned in the pETTRXA-1a/LIC by the ligase independent cloning (LIC) method [57]. Cloning was verified by PCR and both constructions were transformed into *Escherichia coli* BL21(DE3) for protein expression. A His6-tag was fused at the N-terminal to promote purification in a His-Trap-Ni–NTA column (GE Healthcare) for both proteins. Details and specifications of the expression step, strains, and plasmids are described in detail in Additional file 1: Methods section).

### Enzymatic assays

The enzymatic assays were performed according to methodology described in Yang et al. [58]. Briefly, the first reaction, conversion of ferulic acid into feruloyl-CoA, consisted of 100 mM KH_2_PO_4_ buffer (pH 7.0), 2.5 mM MgCl_2_, 1 mM ferulic acid, 2 mM ATP, 0.4 mM coenzyme A, and 1.5 µg of the purified protein FerA. The mixture was incubated at 30 °C for 1 h. Next, the feruloyl-CoA was converted into vanillin by addition of 1.5 µg of the purified protein FerB and incubated for 24 h at the same temperature. Samples were taken from last reaction for GC–MS (gas chromatography–mass spectrometry) quantitative analysis to detect the vanillin production. The substrates and cofactors were purchased from Sigma-Aldrich (St. Loius, MO, USA).

### Analytical methods for vanillin production

To detect phenolics and vanillin production from enzymatic reactions, we used a quantitative GC–MS analysis. The resulting phenolics from enzymatic reactions were extracted by adjusting the pH of the samples to below 2 with 6 mol l^−1^ HCl and addition of butyl acetate (1:1, v:v) and then derivatized [59]. The derivatized samples (1 µl) were analyzed on an Combi-PAL autosampler (Agilent Technologies GmbH, Waldbronn, Germany) coupled to an Agilent 7890 gas chromatograph in split less mode coupled to a Leco Pegasus two time-of-flight mass spectrometer (LECO, St. Joseph, MI, USA) as described by Weckwerth et al. [60]. The chromatograms were exported from the Leco ChromaTOF software (version 3.25) to the R software. Peak detection, retention time alignment, and library matching were performed using the Target Search R-package [61]. Metabolites were quantified by the peak intensity of a selective mass. The intensity normalization procedure was performed by dividing the fresh weight, followed by sum of the total ion count and global outlier replacement [62, 63].

## Results and discussion

### Establishment of lignin-degrading consortium

In Brazil, sugarcane straw is usually returned to the field after harvest. In the present study, the hypothesis under investigation was whether the sugarcane field soil covered by straws, after the plants were harvested, could be a potential source of novel lignolytic enzymes, because it is expected that the microbial population involved in degradation of plant biomass polymers is enriched in this environment (Fig. 1).

According to the previous studies, the establishment of microbial consortia is a powerful strategy to develop an enriched community with a particular metabolic ability, e.g., consortia for lignocellulosic biomass, phenanthrene, and bitumen degradation have been reported [21–23]. Thus, sugarcane field soil was used as inoculum in media containing minimal nutrients together with lignin fragments as the major carbon source. To establish a consortium able to degrade and modify lignin and/or lignin aromatic compounds, a sugarcane soil-derived consortium was adapted and enrichment on lignin medium for up to 50 consecutive weeks.

**Fig. 2:**
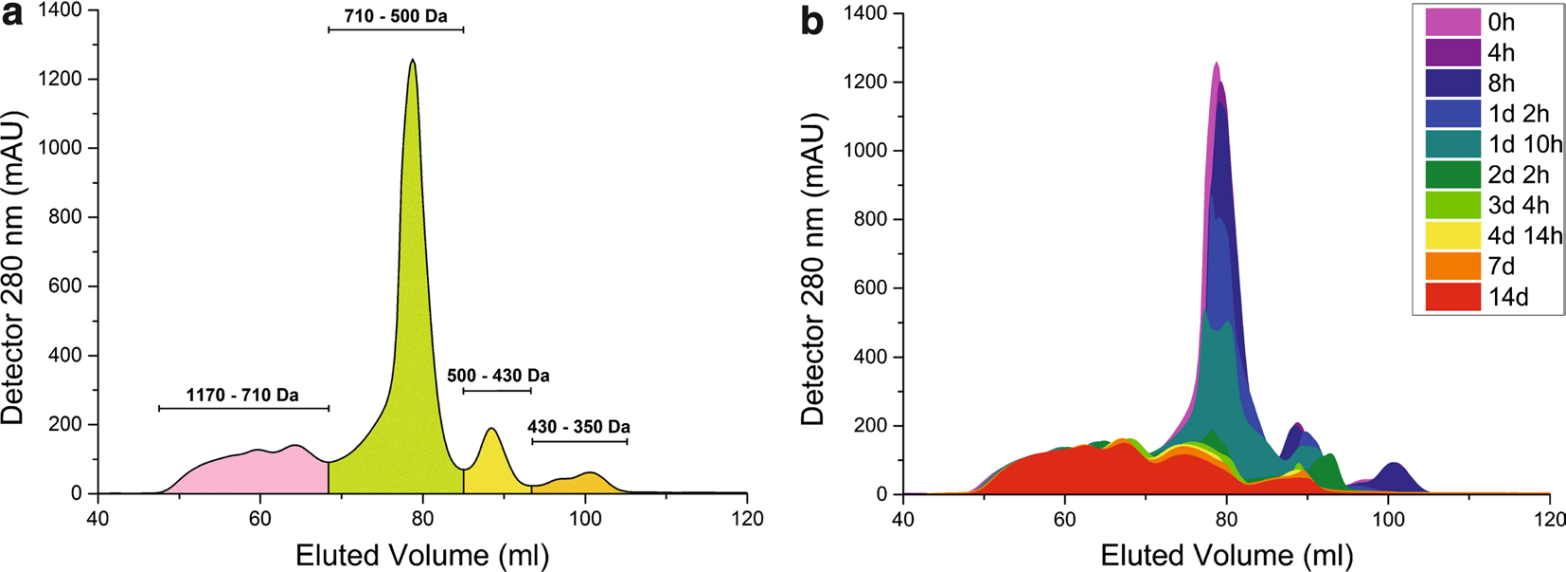
GPC chromatograms showing the molecular weight distribution of **a** lignin-waste stream used as the sole carbon source for establishment of the lignin-degrading consortium (LigMet) and **b** high- and low-molecular-weight fragments detected during 14 days of LigMet cultivation. Above the peaks are the molecular weight distributions in Dalton (Da), which are inversely correlated with the elution time. Different phenolic compounds and polymers were used as internal standards

As illustrated in Fig. 2a, the liquid waste stream used for LigMet cultivation contains lignin fragments ranging from 300 to 1200 Da, including phenolic monomers such as ferulic and cinnamic acid (Additional file 1: Table S1), along with minimal sugar concentrations (450 µM l^−1^). According to Fig. 2b, it is possible to observe that compounds ranging from ~ 700 to ~ 300 Da decreased during the first 34 h of growth, indicating that the consortium was able to use the soluble lignin fragments as a carbon source. Moreover, during the first 40 h, an increase in OD_600_ and consumption of sugars available in the medium was observed, indicating the growth of microbial community (Additional file 1: Figure S1).

### The LigMet structure evaluated through amplicon sequencing

**Fig. 3:**
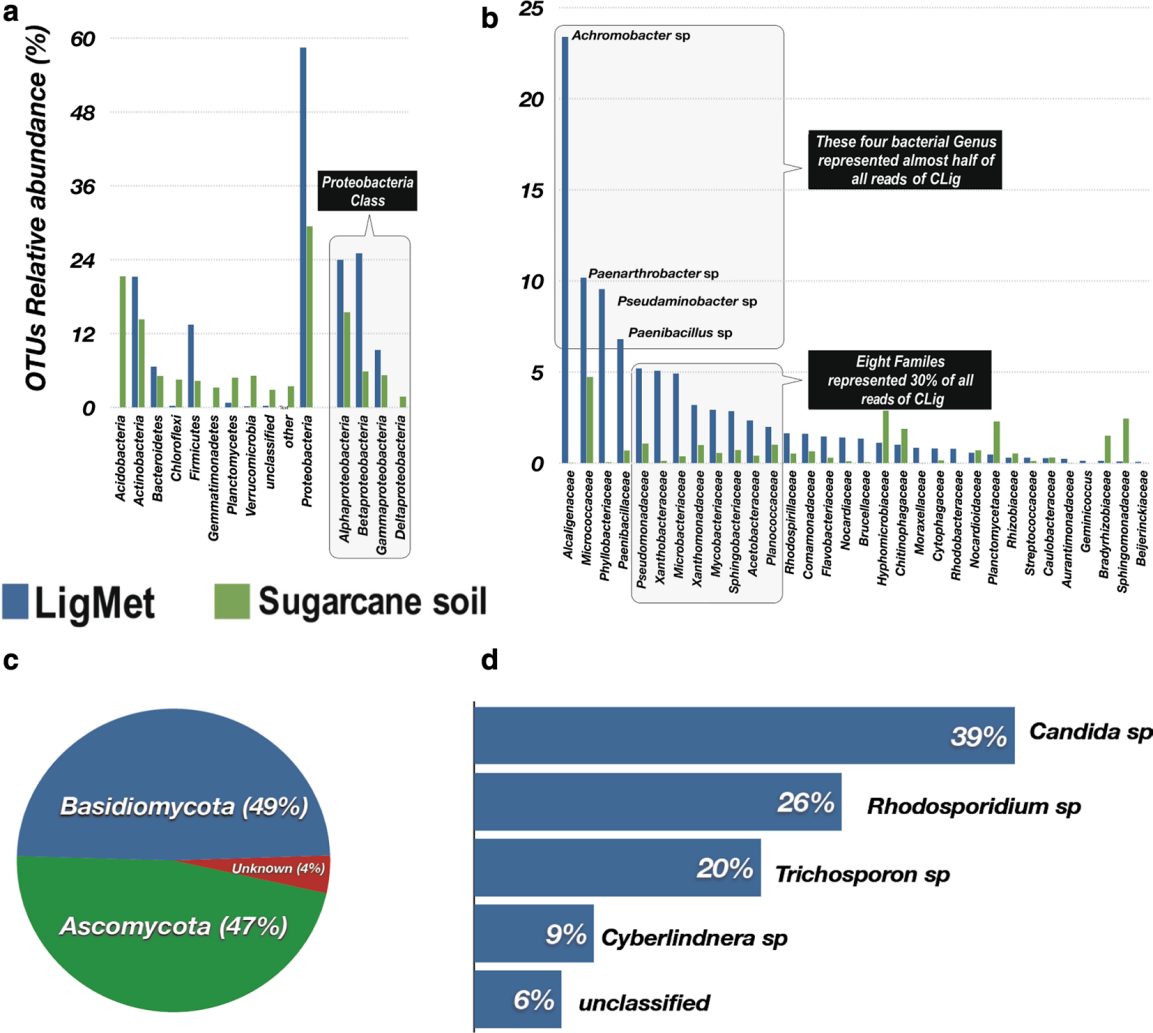
Taxonomic composition of microbial communities from LigMet and the sugarcane soil samples. **a** Bacterial composition at phylum level. **b** Bacterial families of highest abundance in the LigMet and in the sugarcane soil sample. **c** and **d** Fungal communities at phylum and species level, respectively, from LigMet sample based on ITS2 sequencing

Alpha diversity is used to describe microbial community composition of a single sample. The calculated index, which includes species richness and diversity, allows to detect changes in its composition and to compare with other samples. Therefore, the microbial richness and diversity of the sugarcane soil and LigMet were quantified through amplicons sequencing. Statistical data summarizing the sequencing are shown in Additional file 1: Table S2. Clustering of 16S rRNA gene amplicon resulted in 1558 and 355 of OTUs for the sugarcane soil and LigMet, respectively. ACE and Chao1 (richness estimators) and rarefaction analysis suggested that the bacterial species richness in soil and LigMet were entire covered (Additional file 1: Table S3 and Figure S2A, S2B, S3). Similarly, Good’s coverage index of 0.99 for both LigMet and soil indicated that the sequencing was enough to cover the whole bacterial species. Moreover, Shannon and Simpson’s diversity, which calculated species richness and evenness based on different taxa and their relative abundance, were higher for soil (6.3 and 0.99, respectively) in comparison to LigMet (3.4 and 0.92) (Additional file 1: Table S3). The lower microbial diversity, as well as richness and evenness, in enriched consortia is an expected selection response based on selective media [21, 22, 24, 27] and the microorganisms best adapted become dominant [24]. In our study, four OTUs represented 48% of total sequences (Fig. 3b), consequently, decreasing the diversity in LigMet compared to its microbial source (soil).

To compare the taxonomic composition of LigMet and sugarcane soil, the OTU representative sequences were assigned from the phylum to genus level. Figure 3 shows the sugarcane soil and LigMet taxonomic profile. A total of 17 phyla were identified in soil amplicon data and the most abundant phyla were *Proteobacteria* (29% of the total reads), *Acidobacteria* (22%), and *Actinobacteria* (15%) (Fig. 3a). At the class rank, *Alphaproteobacteria* (15%), *Actinobacteria* (14%), and *Acidobacteria* (14%) were predominant in LigMet (Additional file 1: Figure S4). The overall taxonomic profile of the sugarcane soil corroborated with the previous sequencing studies addressing soil microbial communities [64–66].

The LigMet exhibited a significant difference in the relative abundance of the phylogenetic groups compared to the sugarcane soil. The LigMet 16S sequences of *Proteobacteria* origin reached up to 58% of the total sequences classified, which were assigned to the *Betaproteobacteria* (25%), *Alphaproteobacteria* (24%), and *Gammaproteobacteria* (9%) classes (Fig. 3a). Members of *Actinobacteria* and *Firmicutes* were also enriched in LigMet, representing the second and the third predominant phyla with 21% and 13% of the classified sequences, respectively. On the other hand, sequences assigned to *Acidobacteria* class decreased to less than 0.1% in the LigMet, while, in the soil, this group represented around 22% of the total sequences (Fig. 3a). The most abundant bacterial families included *Alcaligenaceae* (24%)*, Micrococcaceae* (11%), *Phyllobacteriaceae* (9%), and *Paenibacillaceaea* (5%), totalizing 48% of the total sequences assigned. Interestingly, the predominant family in LigMet (*Alcaligenaceae*) was virtually non-existent in the soil (less than 0.5%). At the genera level, almost half of total the sequences in the LigMet were assigned to *Achromobacter* (belongs to *Betaproteobacteria*), *Paenarthrobacter (Actinobacteria)*, *Pseudaminobacter* (*Alphaproteobacteria*), and *Paenibacillus (Bacilli).* The result suggests that these microorganisms were key players in LigMet. *Betaproteobacteria*, *Alphaproteobacteria*, *Actinobacteria,* and *Bacilli* classes were frequently reported as degraders lignin and/or lignin-derived aromatic compounds [14, 17, 67, 68]. For example, *Achromobacter* and *Paenarthrobacter* have been characterized for their ability to degrade biphenyl and polycyclic aromatic hydrocarbons, respectively [69, 70]. *Ochrobactrum*, *Rhizobiales*, *Sphingobium,* and *Sphingomonas* are examples of the genus belong to *Alphaproteobacteria* described as lignin-degrading bacteria [14, 18, 69]. Nonetheless, *Pseudomaniobacter* genus is being reported for the first time involved in aromatic compounds degradation.

The presence of fungal community was also analyzed in LigMet. The richness estimators indicated only 12 OTUs, considering an evolutionary distance of 0.03, while Shannon and Simpson diversity were 1.44 and 0.71, respectively (Additional file 1: Table S3). OTUs were assigned to *Basidiomycota* and *Ascomycota* members (Fig. 3c). On average, approximately 94% of all the analyzed sequences could be assigned at the genus level (Fig. 3d). Among them, *Candida* sp. (39%), followed by *Rhodosporidium* sp. (26%), *Trichosporon* sp. (20%), and *Cyberlindnera* sp. (9%), were predominant. *Candida tropicalis* was described as being capable of degrading phenolic compounds [71, 72]. *Trichosporon* and *Cyberlindnera* were reported as dye-decolorizing yeasts [73].

The community had very few sequences assigned to the *Archaea* domain, such as OTUs assigned to the phylum *Thaumarchaeota*, which was identified in sugarcane soil and LigMet with 0.038% and 0.002% of total relative OTU abundance, respectively (Additional file 1: Figure S5). The phylum *Euryarchaeota* was identified only in the LigMet (0.01% relative abundance). Moreover, sequences affiliated with the protozoan class *Litostomatea* were detected in LigMet based on 18S rDNA analyses (Additional file 1: Table S4). According to Simek et al. [74] and Jürgens et al. [75], the taxonomic structure of microbial communities can be shaped by protozoa due to its preferential predation of particular bacterial taxa.

Overall, the statistical analysis showed the enrichment of a few phylogenetic groups in response to adaption of the carbon source added to the medium. As mentioned previously, the taxonomic analysis revealed several microorganisms previously reported as lignin and/or phenolic compounds degraders, thus encouraging further analyses on the genetic content of LigMet.

### Genome assembly and overall functional annotation of LigMet

A total of 97.5 million high-quality paired-end reads (~ 18 gigabase of sequences) were generated from the LigMet sample. Assembled reads resulted in approximately 260 megabases (Mb) of contiguous sequences ≥ 1 Kilobase (Kb). The N50 statistic from the assembled data was 35.5 Kb and the longest contig assembled reached up to 1.3 Mbp. Eukaryotic sequences were not detected in the assembled metagenome, and therefore, downstream analyses focused only on prokaryotic genes and genomes. Considering the complete assembly, 282,237 gene models were predicted (prokaryotic protein-coding regions), where 243,896 of these were larger than 300 bp.

**Fig. 4:**
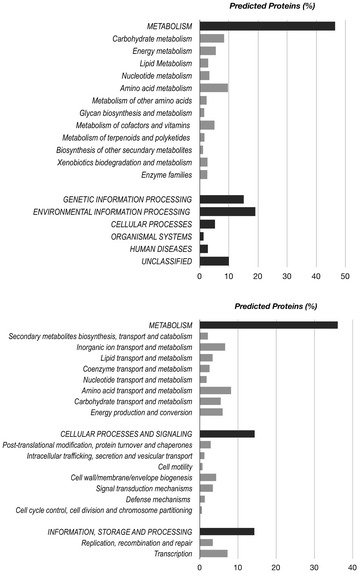
Summary of the annotated predicted proteins from the LigMet Metagenome according to KEGG (**a**) and eggNOG databases (**b**)

**Fig. 5:**
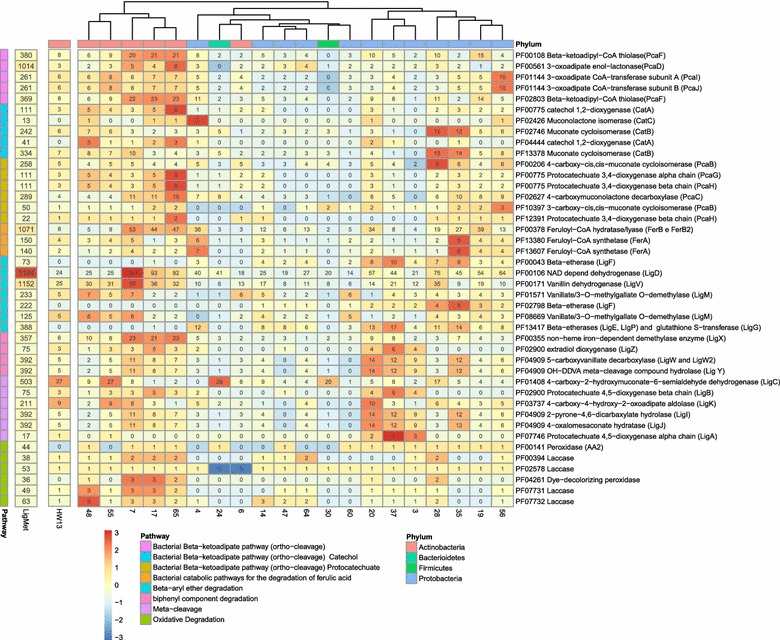
Pfam domains related to aromatic compound degradation pathways. Pfam domains were identified in bacterial draft genomes recovered from LigMet, *Paenarthrobacter* sp. HW13 genome, and in prokaryotic predicted proteins from LigMet

The functional annotation was determined by different methods, where all predicted proteins obtained from the metagenome data set were annotated using EggNOG, KEGG, Pfam, and dbCAN databases. According to the results, 242,299 unigenes (85.8% of the predicted proteins) were assigned based on the EggNOG database, 129,140 (45.7%) based on KEGG, and 208,248 (73.78%) on Pfam, and 8800 (3.1%) had at least one protein domain annotated in dbCAN. The functional annotation obtained based on EggNOG and KEGG was quite similar (Fig. 4); the majority of proteins were classified as belonging to the metabolism category, which harbors a variety of pathways involved in degradation of aromatic compounds. According to the KEGG and Pfam annotation, several aromatic compound degradation pathways were found complete in the metagenome data set, including benzoate degradation (or methyl benzoate) to catechol (or methylcatechol), catechol ortho-cleavage, catechol meta-cleavage, and phthalate degradation (Additional file 1: Figure S6 and Fig. 5). The pathways for metabolism of lignin components and gene designations were described and demonstrated in the previous studies [13, 76–78].

### Reconstruction of draft genomes from the metagenomic data set

The assembly of metagenomic data resulted in the reconstruction of 65 bins (Additional file 1: Table S5), of which 20 were considered draft bacterial genomes, since they presented completeness greater than 75% and contamination less than 10% (Table 1). The taxonomic assignments show that most of the genome bins were assigned to *Alphaproteobacteria* (9 bins), followed by *Actinobacteria* (6 bins) and *Betaproteobacteria* (2 bins). The remaining genome bins were assigned to *Gammaproteobacteria*, *Bacilli*, and *Sphingobacteriia*. These results are in accordance with the previous studies that reported *Rhodococcus*, *Sphingobacterium*, *Ochrobactrum*, *Bacillus*, *Sphingobium,* and *Arthrobacter* as microorganisms that harbor lignolytic enzymes and metabolize aromatic compounds [14, 16, 79]. The 20 draft genomes were further analyzed to depict their metabolic potential on lignin and aromatic compound degradation (Fig. 5).

**Table 1:** Assembly statistics from draft genomes binned from CLig metagenome (20 best results)

Bin Id	Taxonomic affiliation	Completeness (%)	Contamination (%)	Heterogeneity (%)	Total length (bp)	# contigs	# Predicted genes	GC (%)
3	*Sphingomonadaceae*	97.9	2.1	0	2,600,224	14	2487	63.8
4	*Alcaligenaceae*	96.6	3.2	8.3	4,071,016	139	3831	63.9
6	*Microbacteriaceae*	93.0	2.3	25.0	2,775,244	23	2744	70.2
7	*Mycobacterium* sp.	98.6	4.7	7.4	8,113,983	152	7824	67.0
14	*Agrobacterium* sp.	97.0	1.9	0	3,723,529	25	3637	62.4
17	*Rhodococcus* sp.	96.4	1.4	12.5	5,940,093	80	5417	67.9
19	*Rhodobacter* sp.	97.5	3.5	0	4,829,944	102	4636	66.4
20	*Comamonadaceae*	76.1	1.8	0	5,317,169	797	5668	69.5
24	*Sphingobacterium* sp.	92.2	2.2	0	5,342,826	905	5155	40.9
28	*Ochrobactrum* sp.	99.1	10.5	0	8,098,959	173	7885	56.5
30	*Bacillus* sp.	92.6	3.7	0	4,962,376	118	4977	35.4
35	*Acetobacteraceae*	96.7	2.0	0	5,217,664	48	5050	70.2
37	*Sphingobium* sp.	98.1	9.3	5.7	6,313,527	428	6265	59.7
47	*Pseudoxanthomonas* sp.	97.3	1.5	0	3,192,941	22	2886	71.6
48	*Paenarthrobacter* sp.	97.0	4.1	0	4,259,515	57	3930	68.2
55	*Paenarthrobacter* sp.	97.3	1.6	0	4,537,449	80	4185	63.5
56	*Acetobacteraceae*	92.7	1.7	25.0	5,069,773	252	4671	70.4
60	*Hyphomicrobium* sp.	97.9	0.9	0	3,563,628	12	3278	64.7
64	*Methylobacterium* sp.	94.8	1.6	25.0	3,854,043	176	3682	70.1
65	*Rhodococcus*	96.8	1.8	0	5,770,250	74	5292	70.8

### Biocatalysts and pathways involved in lignin degradation and aromatic compound conversion

Pfam-based analysis identified several conserved domains related to lignin and aromatic compound degradation in the LigMet data set and genome bins (Fig. 5). Among these, peroxidases (PF00141), dye-decolorizing peroxidases (PF04261), and laccases (PF00394, PF02578, PF07731, and PF07732) domains, and enzymes that can generate radicals for cleavage of several lignin linkages, were found predominantly in genome bins belonging to the *Actinobacteria* class (bins 48,7,17, and 65) (Fig. 5). These findings corroborated the previous studies, reporting that *Actinobacteria* is able to produce extracellular lignolytic enzymes [14, 17, 80].

Glutathione-dependent beta-etherases of *Sphingobium* sp. were recently described as able to cleave beta-aryl ether bonds of lignin from softwoods and hardwoods [81]. Corresponding domains for the beta-etherases LigE, LigP, LigG, and LigF (considering the PF13417, PF00043, and PF02798) were predominantly found in bins 4, 20, 28, 35, and 37, which were assigned with *Alpha* and *Betaproteobacteria* origin (Fig. 5). Conversion of the beta-aryl ether lignin dimer involves LigEFG and LigD (PF00106) and results in vanillic acid [16], which can also be generated from vanillin by the action of LigV (PF00171). In this pathway, vanillic acid is converted into protocatechuic acid by LigM (PF01571 and PF08669). All these protein domains (LigEFG, LigD, LigV, and LigM) were identified in several bins, indicating their potential to metabolize vanillin and vanillic acid. Nonetheless, they were found predominantly in bins assigned to *Mycobacterium* (bin 7).

The biphenyl linkage is also a common component of lignin structure. Its cleavage is carried out by the cascade pathway LigX (PF0035), LigZ (PF02900), and LigW, LigW2, and LigY (PF04909) [16], also resulting in the central intermediate vanillic acid. With the exception of bins 6 and 28, several *Actinobacteria* genome bins displayed the complete set of proteins needed for biphenyl and vanillic acid conversion (Fig. 5). However, it is possible to notice that this cleavage cascade pathway was identified in only a few bins of *Proteobacteria* origin (20, 37, and 3) (Fig. 5).

The depolymerization of lignin releases a mixture of aromatic monomers that can be used as carbon and energy sources by several microorganisms [82, 83]. Phenolic aromatic monomers can be converted into metabolic intermediates, via catechol and protocatechuate, by the action of dioxygenases, which are classified according to the relative positions of hydroxyl groups (ortho- and meta-cleavage) [13]. The routes can be divided in three blocks: (i) the branch of catechol intermediate (ortho-cleavage), which involves the following enzymes: CatA (PF0775 and PF04444), CatB (PF02746 and PF13378), and CatC (PF02426); (ii) the branch of protocatechuate (meta-cleavage), which involves PcaG (PF00775), PcaH (PF00775 and PF12391), PcaB (PF10397 and PF00206), and PcaC (PF02627); and finally, (iii) the reactions common for both branches, catalyzed by PcaD (PF00561), PcaI (PF01144), PcaJ (PF01144), and PcaF (PF02803 and PF00108). Three *Actinomycetes* genome bins (17, 48, and 65) and two of *Proteobacteria* origin (4 and 20) presented all protein domains related to routes i, ii, and iii. Regarding route (ii), all *Actinobacteria* genome bins and two of *Proteobacteria* origin (bins 20 and 35) were clearly enriched with coding genes involved in protocatechuate degradation. With exception of the *Sphingobacterium* genome bin (24) and the *Bacillus* bin (30), the protein domains corresponding to enzymes related to route iii were mapped in all bins.

The present work disclosed several novel aromatic-degrading enzymes of a high and low degree of homology to previously identified variants. For instance, the BLASTp comparison with beta-etherase (LigE) from *S. paucimobilis* SYK-6 identified 18 orthologous in LigMet, with sequence identity varying from 31 to 64% (E-value from 4e−30 to 0.0). Accordingly, LigMet orthologous to beta-etherase LigF (26 hits), Glutathione S-transferase homolog LigG (3 hits) and beta-etherase LigP (18 hits) from *S. paucimobilis* were recovered as well, with sequence identity varying from 26 to 93% (4e−178 to 9e−11), 29 to 84% (4e−164 to 3e−18), and 31 to 88% (4e−30 to 0.0), respectively.

Moreover, to obtain deeper insight on potential of the microbial community to carry out redox and hydrolytic mechanisms related to lignocellulose degradation, the auxiliary activity (AA) and carbohydrate esterase (CE) families, which act with CAZymes, were predicted based on dbCAN analysis (Additional file 1: Figure S7). The carbohydrate esterases from family 1 (CE1), including feruloyl esterases and *p*-coumaroyl esterases, can break down ester cross links of lignin and hemicelluloses [84, 85]; and glucuronyl esterases (CE15) were reported to catalyze ester linkage hydrolysis between glucuronoxylan and lignin [86]. Furthermore, acetyl esterases (described in families CE01, 02, 03, 04, 05, 06, 07, 12, and 16) play a role in degradation of xylose units esterified with acetic acid [85]. Nonetheless, only the families CE1 and CE4 were predominant in the LigMet metagenome data set and widespread in all genome bins (Additional file 1: Figure S7 and S8). Similar to CE families, AA were abundant in the LigMet metagenome, notably the AA3 family members, which may be related to peroxidase activity, thus corroborating with the previous PFAM-based functional analysis.

### *Paenarthrobacter* sp. HW13 genome

To complement the metagenomic discovery approach, the isolation of a novel lignin-degrading microorganism from LigMet was performed using HW or LW as the sole carbon source in media. Among the obtained colonies, only a single strain cultivated on HW exhibited a decolorization halo after being covered with an agar solution supplemented with Azure-B dye. Based on the 16S rRNA gene comparison, the analysis revealed that the strain HW13 shares 96.8% 16S rRNA gene sequence similarity with *P. ureafaciens* DSM 20126^T^ as the closest related species with a valid nomenclature (Additional file 1: Figure S9). Therefore, the strain may represent a new species within the *Paenarthrobacter* genus. The strain was denominated *Paenarthrobacter* sp. HW13.

**Fig. 6:**
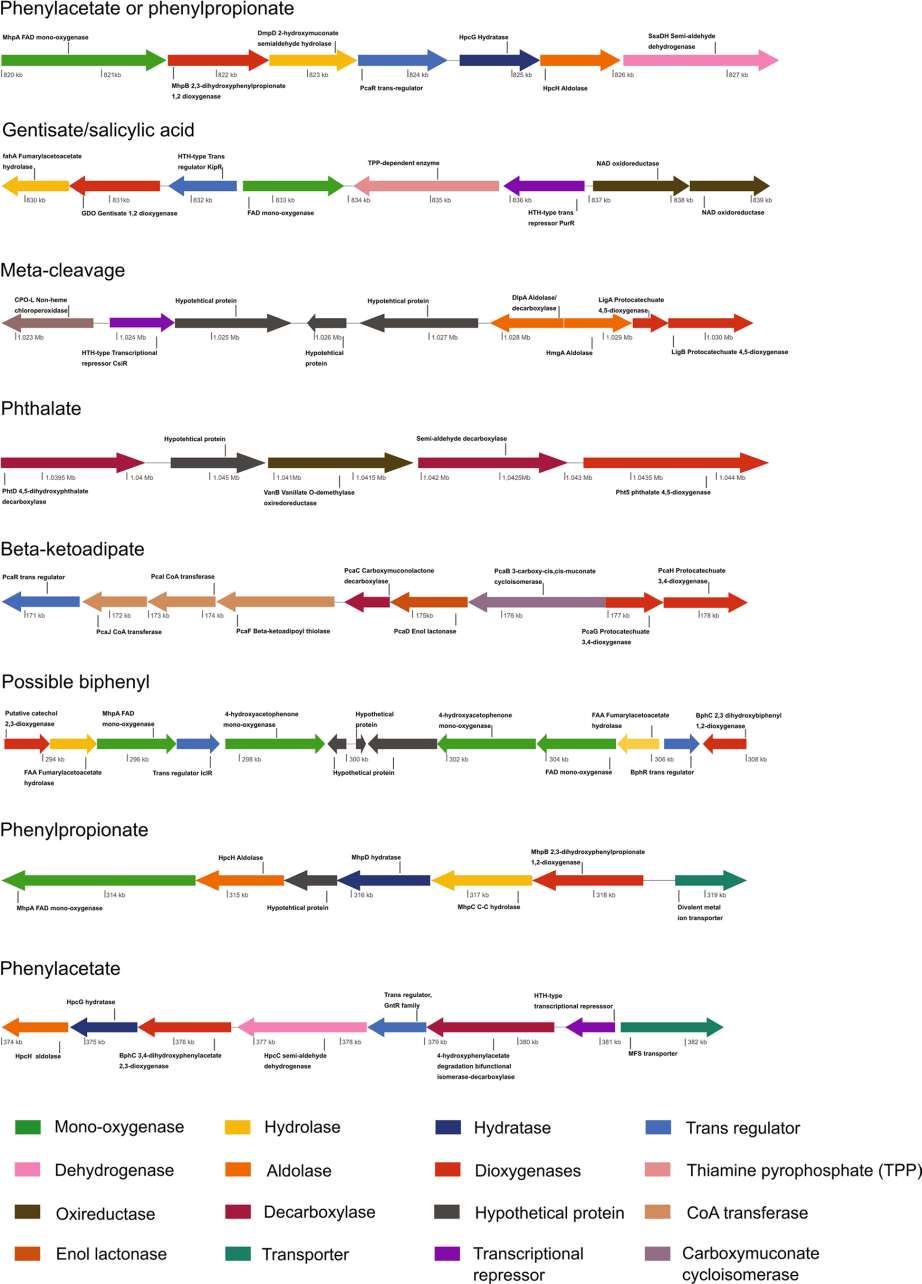
Schematic representation of gene clusters involved in aromatic degradation pathways identified in the *Paenarthrobacter* sp. HW13 genome. Homologous genes are shown in the same color and the predicted gene products are indicated

The genome sequence analysis of the strain HW13 depicted its potential to metabolize lignin fragments and phenolic compounds (Fig. 6). Two libraries were sequenced on an Illumina/MiSeq system, resulting in 3,746,638 pair-end reads and 1,671,986 mate-pair reads, accounting for 1083 Mb. After data processing, the assembly resulted in three contigs consisting of 4,091,031 bases and featuring a CG content of 63%. Additional details of the genome features can be found in Additional file 1: Table S6. To determine whether strain HW13 correspond to bin 48 or 55 (both assigned to the *Paenarthrobacter* genera), the microbial species identified (MiSi) method [87] was conducted, as implemented in IMG/ER. The P probability of bins 48 and 55 being assigned to the same species as HW13 was 0.0 and 0.99, respectively. Moreover, strain HW13 showed 100% 16S rRNA gene sequence identity to the 16S rRNA gene found in genome bin 55. The genomic analysis confirmed the prediction of several previously identified genetic determinants involved in aromatic compound metabolism, including the protocatechuate, catechol, phenylacetate, gentisate, and phenylpropionate degradation pathways (Fig. 6).

### Biosynthesis of vanillin from ferulic acid

**Fig. 7:**
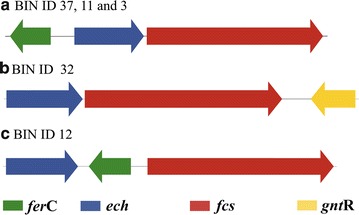
Structure of the F*erA* and *FerB* genes clusters identified in the LigMet metagenome. The structure of *FerA* and *FerB* genes clusters described in **a** *Sphingobium* sp. and **b** *Bacillus subtilis*, in which the regulator gene belongs to the GntR family, and **c** *Pseudomonas* sp., showing different arrangements of the regulator (*FerC*). The three gene cluster configurations were identified in the LigMet [17–19], as well as on draft genomes recovered from LigMet, as indicated. Homologous genes are shown in the same color and the predicted gene products are indicated

The occurrence of aromatic conversion pathways in LigMet to produce high value chemicals was validated through biochemical vanillin production. Ferulic acid is found in lignocellulosic biomass, and it is a precursor for biovanillin production [88]. The conversion of ferulic acid into vanillin has been reported in several microorganisms via coenzyme A-dependent, a non-beta-oxidative pathway [58, 89–91], including feruloyl-CoA synthetase (FerB) and enoyl-CoA hydratase/aldolase, (FerB) [92], which are regulated by *ferC*, an MarR-type transcriptional regulator [93]. In this study, the protein domains related to FerA (PF13380) and FerB (PF00378) were found in several bins described in Fig. 5. The clusters of genes related to ferulic acid conversion into vanillin were manually identified in the LigMet, two presenting similar genetic structures previously described in *P. fluorescens* BF13 [93] and *S. paucimobilis* SYK-6 [78] (Fig. 7). In addition, another gene cluster was found in LigMet of similar organization to the gluconate operon of *B. subtilis* [94], which is regulated by a GntR family protein (Fig. 7).

Candidates’ genes for *ferA* (derived from bin 3) and *ferB* (derived from bin 11) were selected for further analyses. The FerA_B3 (bin 3) when compared to other bacterial feruloyl-CoA synthetases displays 60% amino acid identity to *S. paucimobilis* (A0A031JK39), followed by *Sphingobium* sp. (60%; BAK67177), *Delftia acidovorans* (55%; CAC83622), and *Burkholderia glumae* (52%; ACR31088). The FerB_B11 (bin 11) compared to other bacterial enoyl-CoA hydratases shows 63% amino acid identity to *Pseudomonas nitroreducens* (C3VA24), followed by *Amycolatopsis methanolica* (59%; A0A076MUC3), *Streptomyces* sp. (55%; S5LPF1), and *D. acidovorans* (55%; Q8VNW7). Amino acid sequences from NCBI database and Uniprot with similarity higher than 20% to FerA_B3 and FerB_B11 were considered to construct phylogenetic trees (Additional file 1: Figure S10). It evidenced the relationship FerA_B3 and FerB_B11 with other bacterial feruloyl-CoA synthetases and enoyl-CoA hydratases, respectively, but the low bootstrap value at most of the clades denoted the high divergence of FerA_B3 and FerB_B11.

**Fig. 8:**
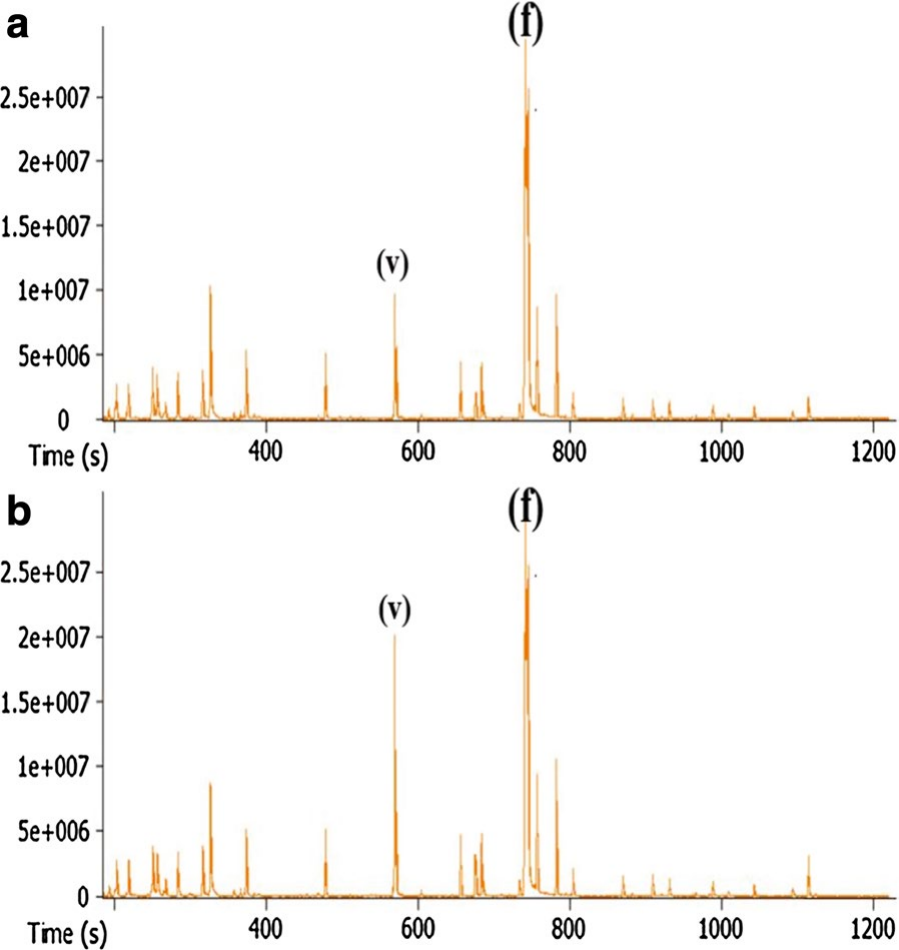
GC–MS chromatograms of products from the reaction of ferulic acid into vanillin by the enzymes FerA and FerB. The *Y*-axis represents relative abundance (ion count) and the *X*-axis represents retention time (in seconds). Vanillin and ferulic acid peaks are depicted in chromatograms A and B by the letters (*v*) and (*f*), respectively. **a** 6 h reaction; **b** 24 h reaction

For the bioproduction of vanillin, candidates genes for *ferA* and *ferB* were then synthesized and successfully cloned for expression in *E. coli,* followed by purification by liquid chromatography. The conversion of ferulic acid into vanillin, after combining the two purified enzymes, was confirmed by GC–MS. The vanillin production from ferulic acid was detected after 6 and 24 h incubation using the purified recombinant proteins (Fig. 8). The biochemical and structural characterization of the two proteins, FerA_B3 and FerB_B11, will be the focus for future studies.

## Conclusions

Different approaches were applied to validate the establishment of a lignin-degrading consortium and applicability of the metagenomic strategy. From the analysis of massive DNA sequencing data, several microorganisms and enzymes linked to lignin-degrading metabolic pathways were identified in LigMet. Furthermore, gene clusters involved in the non-beta-oxidative pathway for vanillin production were depicted, followed by production of recombinant FerA and FerB in functional form for successful production of the phenolic aldehyde. The genome sequencing analysis of a bacterium revealed eight gene clusters encoding proteins related to aromatic degradation, suggesting potential for biotechnological application.

The LigMet metagenome represents a vast reservoir of genes coding for enzymes involved in lignin depolymerization and assimilation. The targeted metagenomic discovery platform described in the present study is of potential interest to reveal optimized gene sets and microorganisms for initiatives based on synthetic biology principles to produce high value chemicals from lignocellulose. The straightforward metagenomic strategy described could also be applied to other fields such as the development of antibiotic producing microorganisms or the recycling of plastic polymers.

## Additional file

**Additional file 1: Table S1.** Compounds identified by gas chromatography–mass spectrometry (GC-MS) in the lignin-waste stream used for establishment of the lignin-degrading microbial community (LigMet). **Table S2.** Sequencing statistics and data processing of amplicon libraries constructed for profiling LigMet and soil samples analyzed. **Table S3.** Diversity and richness indices of the LigMet and soil samples based on 16S rRNA and ITS2 region sequences. **Table S4.** Protozoa identified in LigMet based on 18S rRNA sequencing. **Table S5.** Assembly statistics from draft genomes recovered from LigMet (all assemblies). **Table S6.** Genome statistics of *Paenarthrobacter* sp. str. HW13. **Figure S1.** Microbial growth was monitored by OD 600 nm, observing exponential growing during the first 40 hours of consortium growth. The consumption of reducing sugars over time as monitored by DNS, the exponential phase was completed after the first 40 hours of growth when monitoring sugar consumption. **Figure S2.** Rarefaction curves based on targeted sequencing of 16S rRNA gene amplicons derived from the LigMet (**A**) and sugarcane soil (**B**) samples. The rarefaction curves of each biological replicate are shown in different colors. **Figure S3.** Rarefaction curves based on targeted sequencing of the ITS2 region derived from the LigMet sample. The rarefaction curves of each biological replicate are shown in different colors. **Figure S4.** The taxonomic profiles from LigMet and sugarcane soil samples at the class level based on 16S rRNA gene amplicon. The respective relative abundances of each biological replicate for LigMet and sugarcane soil are shown. **Figure S5.** The archaeal phylum abundance in LigMet and sugarcane soil sample. The relative abundance is shown in percentage for each biological replicate of the LigMet and sugacarcane soil. **Figure S6.** Metabolic pathways related to aromatic compound degradation identified in LigMet according to KEGG automatic annotation. **Figure S7.** Classification of the predicted proteins from the LigMet according to the dbCAN database. **Figure S8.** Predicted auxiliary activity (AA) and carbohydrate esterase (CE) families from LigMet and draft genomes, based on the dbCAN database. AA and CE families are related to peroxidase activity and break down of lignin ester cross links, respectively. **Figure S9.** Phylogenetic relationship of the strain HW13 relative to the most closely related strains of the genus *Paenarthrobacter*. EzBioCloud webserver was used to perform a similarity-based search of HW13 16S rRNA to retrieve the most closely related sequences. The resulting 16S rRNA sequences were aligned using the MAFFT v7.299b software. A phylogenetic tree was inferred using the maximum likelihood method implemented in RAxML v8.2.0, evolutionary distances were based on the GTRGAMMAI model, inferred as the best model by jModelTest2. Numbers at the nodes are percentages of bootstrap values obtained by repeating the analysis 1,000 times. The type strains are marked with a superscript ‘T’. Accession numbers are shown in parentheses. **Figure S10.** Phylogenetic relationships among feruloyl-CoA synthetase (upper) and Enoyl-CoA hydratase/aldolase. The phylogenetic tree was generated using amino acid sequences retrieved from NCBI and Uniprot database. The sequences were aligned using MAFFT v7.299b software [5]. The phylogenetic tree was reconstructed using maximum likelihood method implemented in RAxML v8.2.0 [6], evolutionary distances were based on the GTRGAMMAI model, inferred as the best model by jModelTest2 [7]. The bootstrap values (1,000 replicate runs, shown as %) higher than 70 % are represented. Accession numbers are listed in parentheses. The FerA_B3 and FerB_B11 amino acid sequence retrieved from LigMet data set is printed in bold.

## Abbreviations

LigMet: lignin-degrading consortium
DNS: 3,5-dinitrosalicylic acid
PCR: polymerase chain reaction
16S rDNA: 16S rRNA gene sequence
ITS: nuclear ribosomal internal transcribed spacer
OTU: operational taxonomic unit
BLAST: Basic Local Alignment Search Tool
RDP database: ribosomal database project database
Pfam: protein families
dbCAN: database for carbohydrate-active enzyme annotation
KEGG: Kyoto encyclopedia of genes and genomes
eggNOG: evolutionary genealogy of genes: Non-supervised Orthologous Groups
MiSi: microbial species identified
HW: high molecular weight
CAZyme: carbohydrate-active enzyme
GH: glycoside hydrolase
AA: auxiliary activities
CE: carbohydrate esterase
HMM: hidden Markov model
GC–MS: gas chromatography–mass spectrometry
