# Supplementary material for: Lignolytic-consortium omics analyses reveal novel genomes and pathways involved in lignin modification and valorization
Source: Biotechnol Biofuels. 2018 Mar 22;11:75. doi: 10.1186/s13068-018-1073-4 (PMC5863372; doi:10.1186/s13068-018-1073-4)
Supplement: Supplementary file 1 — Additional file 1: Table S1. Compounds identified by gas chromatography–mass spectrometry (GC-MS) in the lignin-waste stream used for establishment of the lignin-degrading microbial community (LigMet). Table S2. Sequencing statistics and data processing of amplicon libraries constructed for profiling LigMet and soil samples analyzed. Table S3. Diversity and richness indices of the LigMet and soil samples based on 16S rRNA and ITS2 region sequences. Table S4. Protozoa identified in LigMet based on 18S rRNA sequencing. Table S5. Assembly statistics from draft genomes recovered from LigMet (all assemblies). Table S6. Genome statistics of Paenarthrobacter sp. str. HW13. Figure S1. Microbial growth was monitored by OD 600 nm, observing exponential growing during the first 40 hours of consortium growth. The consumption of reducing sugars over time as monitored by DNS, the exponential phase was completed after the first 40 hours of growth when monitoring sugar consumption. Figure S2. Rarefaction curves based on targeted sequencing of 16S rRNA gene amplicons derived from the LigMet (A) and sugarcane soil (B) samples. The rarefaction curves of each biological replicate are shown in different colors. Figure S3. Rarefaction curves based on targeted sequencing of the ITS2 region derived from the LigMet sample. The rarefaction curves of each biological replicate are shown in different colors. Figure S4. The taxonomic profiles from LigMet and sugarcane soil samples at the class level based on 16S rRNA gene amplicon. The respective relative abundances of each biological replicate for LigMet and sugarcane soil are shown. Figure S5. The archaeal phylum abundance in LigMet and sugarcane soil sample. The relative abundance is shown in percentage for each biological replicate of the LigMet and sugacarcane soil. Figure S6. Metabolic pathways related to aromatic compound degradation identified in LigMet according to KEGG automatic annotation. Figure S7. Classification of the predicted pr [file 13068_2018_1073_MOESM1_ESM.pdf]

Lignolytic-consortium omics analysis reveal novel genomes and pathways involved in lignin modification and valorization

*Eduardo C. Moraes[1¶], Thabata M. Alvarez[2¶], Gabriela F. Persinoti[1¶], Geizecler Tomazetto[1], Livia B. Brenelli[1], Douglas A. Paixão[1], Gabriela Cristina Ematsu[1], Juliana A. Aricetti[1], Camila Caldana[1], Neil Dixon[3], Timothy D. H. Bugg[4], Fabio M. Squina[5]**

¶ *Contributed equally*

This PDF file includes:

Materials and Methods

Tables S1 to S6

Figs. S1 to S10.

References

Methods

Cloning, expression and purification of genes involved in bioconversion of ferulic acid into vanillin production

LigMet metagenomic dataset was screened based on Pfam families (PF13380 and PF00378) to find the candidate sequences coding feruloyl coenzyme A synthetase and enoyl-CoA hydratase. Genes *fer*A_B3 and *fer*B_B11*,* encoding feruloyl coenzyme A synthetase and enoyl-CoA hydratase, respectively, were employed in the biotransformation of ferulic acid into vanillin. The sequences were synthetized by Biomatik (Biomatik Corporation, Canada) and inserted into the pET28a-vector and transformed into *Escherichia coli* BL21(DE). The *fer*A_B3 gene sequence was sub-cloned in the pETTRXA-1a/LIC by the ligase independent cloning (LIC) method [1]. The resulting plasmids were then transformed into *E. coli* BL 21 (DE) for expression. The recombinant *E. coli* were grown at 37 ºC in Luria Bertani medium containing 100 mg/L kanamycin. When the culture reached an optical density (OD_{600} nm) of 0.6~0.8, IPTG was added to the culture at a final concentration of 0.5 mM. Thus, the culture was incubated for 4 hours at 30 º C for over-expression. Cells were harvested by centrifugation and suspended in bidding buffer (20 mM sodium phosphate pH 7.4, 100 mM sodium chloride and 5mM imidazole) supplemented with phenylmethanesulfonyl fluoride (PMSF) at final concentration of 1 mM and lysozyme at final concentration of 0.5 mg/mL. Cells were incubated for 30 minutes on ice and then lysed by sonication. Soluble fractions were obtained by centrifugation at 14,000 x g at 4 ºC for 30 minutes and then loaded in His-Trap-Ni-NTA columns (GE Healthcare), pre-equilibrated with binding buffer. Recombinant proteins were eluted with an elution buffer (20 mM Tris-HCl, pH 8.0, 100 mM NaCl, 400 mM imidazole). Eluted fractions were evaluated by sodium dodecyl sulfate-polyacrylamide gel electrophoresis (SDS-PAGE) and protein concentrations were determined according to the Bradford method. Purified proteins were stored at - 80 ºC until further use.

Table S1. Compounds identified by gas chromatography–mass spectrometry (GC-MS) in the lignin-waste stream used for establishment of lignin-degrading microbial community (LigMet).

Retention Time (min)	Match (%)	Compound
9.739	87.6	Butanoic acid
10.241	96.1	Latic acid
12.235	93.8	Butanoic acid
12.995	94.3	3-Hydroxypropanoic acid
13.218	86.8	Butyric acid, 3-hydroxy
13.991	92	Oxalic acid
14.29	73.8	Pentenoic acid, 4-[(trimethylsilyl)oxy]-, trimethylsilyl ester
15.192	80	Pentenoic acid, 4-[(trimethylsilyl)oxy]-, trimethylsilyl ester
15.857	92.1	Glycerol
17.511	79.7	Propanoic acid, 2-methyl-2,3-bis[(trimethylsilyl)oxy]-, trimethylsilyl ester
18.366	81.2	Glyceric acid
18.78	75.5	Succinic acid
21.744	84.8	2(3H)-Furanone, dihydro-3-[(trimethylsilyl)oxy]-3-[[(trimethylsilyl)oxy]methyl]-
25.209	73	Butyric acid, 4-hydroxy
25.901	75.8	Butyric acid, 4-hydroxy
27.251	80.5	Arabinoic acid, 2,3,5-tris-O-(trimethylsilyl)-, ç-lactone, l-
28.221	81.6	D-Erythro-Pentonic acid, 3-deoxy-2,5-bis-O-(trimethylsilyl)-2-C-[[(trimethylsilyl)oxy]methyl]-, ç-lactone
28.621	82.6	D-Erythro-Pentonic acid, 3-deoxy-2,5-bis-O-(trimethylsilyl)-2-C-[[(trimethylsilyl)oxy]methyl]-, ç-lactone
31.212	61	Acetic acid, [4-methoxy-3-(trimethylsiloxy)phenyl]-, methyl ester
33.192	93.2	Cinnamic acid, 4-hydroxy-, trans-
36.393	76.6	Trans-ferulic acid

Legend. The lignin source used in this study was a soluble stream generated by pilot-scale steam explosion and alkaline delignification of sugar cane bagasse [2]. The lignin profiling was performed with derivatized samples by a gas chromatography–mass spectrometry (GC-MS) system (Agilent GC 6890 and MSD 5973N series, Agilent, USA), according to Suguiyama et al [3]. The peaks were identified and quantified in comparison with authentic standards and the NIST Mass Spectral Library. The peak retention time and confidence level in sample identification with GC-MS are presented.

Table S2. Sequencing statistics and data processing of amplicons libraries constructed for profiling of the LigMet and soil sample analyzed.

Gene amplicon/Target primers/Replicate	Raw sequences[1]	Sequences Trimming/Merged/Chimeras[2]	Average amplicon size [bp]	OTUs[3]
LigMet				
16S rRNA/*Bacteria*/A	744,353	310,123	292	353
16S rRNA/*Bacteria*/B	519,315	212,420	292	343
16S rRNA/*Bacteria*/C	519,302	206,284	292	355
Soil				
16S rRNA/ *Bacteria /A*	42,349	20,193	292	1,419
16S rRNA/ *Bacteria /A*	101,080	52,903	292	1,551
16S rRNA/ *Bacteria /A*	137,133	77,397	292	1,558
LigMet				
ITS2/ *Fungi* /A	51,188	41,600	337	11
ITS2/ *Fungi* /B	40,950	35,014	331	12
ITS2/ *Fungi* /C	52,795	40,914	334	9

[1] Total counting of paired-end reads.
[2] After quality filtering of reads, merged sequences and removal of chimeric sequences.
[3] Number of Operational Taxonomic Units (OTUs).

Table S3. Diversity and richness indices of LigMet and soil samples based on 16S rRNA and ITS2 region sequences.

Target region / Replicate	Chao1	se.chao1	ACE	se.ACE	Shannon	Simpson	InvSimpson
16S Soil1	1503.00	17.18	1497.26	17.85	6.19	0.99	158.94
16S Soil2	1552.12	1.24	1554.16	19.35	6.36	1.00	201.28
16S Soil3	1558.07	0.28	1558.55	17.67	6.34	0.99	184.25
16S LigMet1	354.74	1.71	357.49	9.26	3.39	0.92	12.38
16S LigMet2	347.36	2.89	353.83	9.15	3.40	0.92	12.56
16S LigMet3	338.09	2.39	342.42	9.08	3.39	0.92	12.51
ITS2 LigMet1	11	0.16	12.06	1.64	1.46	0.73	3.70
ITS2 LigMet2	12	0.48	12.37	1.68	1.45	0.71	3.47
ITS2 LigMet3	9	0.00	9.00	0.94	1.44	0.70	3.35

Table S4. Protozoa identified in LigMet based on 18S rRNA sequencing.

Clones	Taxonomic affiliation	Score	Query Cover	E.value	Ident[1]	Accession[2]
1.6	*Metadinium minorum*	895	88%	0.0	99%	JN116224.1
1.10	*Metadinium minorum*	917	88%	0.0	99%	JN116224.1
2.4	*Metadinium medium*	904	91%	0.0	99%	JN116208.1
2.9	*Metadinium minorum*	922	88%	0.0	100%	JN116224.1

Legend. Fragments of 18S rRNA gene were amplified using primers [4] and total DNA from LigMet as templates. The PCR products were purified, cloned in pGEM-TEasy Vector, and transformed in *Escherichia coli* DH5α. Clones were sequenced with Sanger-sequencing technology, according to the manufacturer's instructions. Sequences were compared with references sequences in the public databases GenBank using BLASTn tool.

[1] Best Blast hit reference nucleotides sequences from NCBI database.

[2] GenBank accession numbers.

Table S5. Assembly statistics from draft genomes recovered from LigMet (all assemblies).

Bin Id	Taxonomy	Completeness (%)	Contamination (%)	Heterogeneity (%)	Total length (bp)	contigs	Predicted genes	GC (%)
**0**	N/A	0.00	0.00	0.00	1.008	1	1	67.86
**1**	N/A	0.00	0.00	0.00	347.543	168	395	33.47
**2**	*Burkholderiales*	15.60	0.39	100.00	1.450.957	664	1791	70.95
**3**	*Sphingomonadales*	97.85	2.10	0.00	2.600.224	14	2487	63.85
**4**	*Betaproteobacteria*	96.64	3.15	8.33	4.071.016	139	3831	63.90
**5**	N/A	0.00	0.00	0.00	3.902	3	3	72.60
**6**	*Actinomycetales*	93.03	2.30	25.00	2.775.244	23	2744	70.21
**7**	*Actinomycetale*	98.15	4.70	7.41	8.113.983	152	7824	67.03
**8**	N/A	0.00	0.00	0.00	1.703	1	1	74.99
**9**	*Bacteria*	65.96	29.90	22.00	4.259.062	2507	5745	71.51
**10**	*Burkholderiales*	65.56	57.81	69.51	4.042.450	1865	4847	67.40
**11**	*Alphaproteobacteria*	96.56	0.19	50.00	2.670.248	112	2600	67.01
**12**	*Rhizobiales*	96.97	79.92	2.96	6.835.854	532	6769	66.99
**13**	N/A	0.00	0.00	0.00	3.010	2	2	68.74
**14**	*Rhizobiales*	97.00	1.88	0.00	3.723.529	25	3637	62.37
**15**	*Bacteria*	100.00	5.96	0.00	4.073.576	105	3540	39.91
**16**	*Bacteria*	95.30	84.12	46.15	8.685.285	96	8342	64.68
**17**	*Actinomycetales*	96.42	1.35	12.50	5.940.093	80	5417	67.93
**18**	*Bacteria*	75.09	67.76	13.33	3.651.647	1656	4885	70.57
**19**	*Rhodobacteraceae*	97.47	3.53	0.00	4.829.944	102	4636	66.36
**20**	*Burkholderiales*	76.05	1.77	0.00	5.317.169	797	5668	69.45
**21**	N/A	0.00	0.00	0.00	1.075	1	1	56.19
**22**	*Rhizobiales*	91.16	2.39	42.86	3.339.285	178	3466	64.59
**23**	*Rhizobiales*	97.58	132.77	12.48	7.677.502	928	7988	64.43
**24**	*Bacteroidetes*	92.20	2.22	0.00	5.342.826	905	5155	40.87
**25**	N/A	0.00	0.00	0.00	1.316	1	1	63.75
**26**	*Bacteria*	4.02	0.00	0.00	785.243	366	929	56.40
**27**	*Rhizobiales*	43.20	12.76	10.23	2.498.284	1672	3453	68.27
**28**	*Alphaproteobacteria*	99.09	10.50	0.00	8.098.959	173	7885	56.49
**29**	N/A	0.00	0.00	0.00	1.544	1	1	58.87

**30**	*Bacillaceae*	92.62	3.65	0.00	4.962.376	118	4977	35.39
**31**	N/A	0.00	0.00	0.00	38.220	22	31	64.11
**32**	*Actinomycetales*	97.87	4.23	28.57	7.050.587	101	6810	67.08
**33**	N/A	0.00	0.00	0.00	1.005	1	2	38.81
**34**	*Bacteria*	99.37	268.86	8.94	19.268.557	1141	18967	62.86
**35**	*Rhodospirillales*	96.72	1.99	0.00	5.217.664	48	5050	70.22
**36**	N/A	0.00	0.00	0.00	18.311	15	13	44.24
**37**	*Sphingomonadales*	98.08	9.27	5.66	6.313.527	428	6265	59.70
**38**	*Bacteria*	97.77	318.77	39.02	16.058.880	881	15609	70.68
**39**	*Rhodospirillales*	94.41	78.08	43.04	10.169.992	724	9860	67.73
**40**	N/A	0.00	0.00	0.00	10.000	1	1	71.56
**41**	N/A	0.00	0.00	0.00	1.049	1	1	68.64
**42**	*Bacteria*	25.86	154.26	8.01	1.201.784	543	1420	65.79
**43**	*Bacteria*	48.33	13.31	9.09	2.081.579	1291	2700	42.00
**44**	N/A	0.00	0.00	0.00	3.241	3	2	49.34
**45**	N/A	0.00	0.00	0.00	1.859	1	2	63.26
**46**	*Bacteria*	85.55	82.24	58.33	7.703.438	167	6796	69.33
**47**	*Xanthomonadaceae*	97.33	1.46	0.00	3.192.941	22	2886	71.25
**48**	*Micrococcaceae*	96.96	4.12	0.00	4.259.515	57	3930	68.16
**49**	*Bacteria*	89.05	0.47	0.00	4.183.797	1099	4424	62.61
**50**	*Alphaproteobacteria*	97.76	1.83	50.00	3.596.013	141	3525	67.47
**51**	*Actinomycetales*	90.37	1.68	27.27	6.098.250	277	5994	68.00
**52**	*Bacteria*	12.41	0.00	0.00	2.264.077	1256	2977	65.64
**53**	N/A	0.00	0.00	0.00	1.370	1	-	40.51
**54**	*Bacteria*	73.28	78.79	9.27	7.202.492	3594	9569	64.18
**55**	*Micrococcaceae*	97.31	1.59	0.00	4.537.449	80	4185	63.52
**56**	*Rhodospirillales*	92.74	1.66	25.00	5.069.773	252	4671	70.35
**57**	*Bacteria*	27.74	13.79	87.50	9.205.553	1115	9326	68.01
**58**	N/A	0.00	0.00	0.00	10.000	1	10	63.64
**59**	*Burkholderiales*	97.97	116.83	30.02	15.353.582	830	14711	67.27
**60**	*Rhizobiales*	97.93	0.86	0.00	3.563.628	12	3278	64.65
**61**	N/A	0.00	0.00	0.00	45.300	16	36	73.91

**62**	N/A	0.00	0.00	0.00	3.426	2	2	69.09
**63**	N/A	0.00	0.00	0.00	235.900	112	179	59.75
**64**	*Rhizobiales*	94.80	1.62	25.00	3.854.043	176	3682	70.11
**65**	*Actinomycetales*	96.80	1.75	0.00	5.770.250	74	5292	70.80
**66**	N/A	0.00	0.00	0.00	7.662	3	4	70.74

Table S6. Genome statistics for *Paenarthrobacter* sp. HW13

Feature	Chromosome
Total length (bp)	4,091,031
Scaffold	3
Largest contig (bp)	2,064,645
GC (%)	63.43
Total number of genes	3,797
Protein coding genes	3,731
Protein coding genes with function prediction	3,033
rRNA operons	6
tRNAs	52
Gene completeness	99.71%

SUPPLEMENTARY FIGURE CAPTIONS

Fig. S1. Microbial growth was monitored by OD 600 nm, observing exponential growing during the first 40 hours of consortium growth. The consumption of reducing sugars over time was monitored by DNS, and the exponential phase was completed after the first 40 hours of growth when monitoring sugar consumption.

Fig. S2. Rarefaction curves based on target sequencing 16S rRNA gene amplicons derived from LigMet (**A**) and sugarcane soil (**B**) samples. The rarefaction curves of each replicate are shown in different color.

Fig. S3. Rarefaction curves based on target sequencing ITS2 region derived from LigMet sample. The rarefaction curves of each biological replicate are shown in different color.

Fig. S4. The taxonomic profile of LigMet and sugarcane soil at class level based on 16S rRNA gene amplicon. The respective relative abundances of each biological replicate for LigMet and sugarcane soil are shown.

Fig. S5. The archaeal phylum abundance in LigMet and sugarcane soil sample. The relative abundance is shown in percentage for each biological replicate of the LigMet and sugacarcane soil.

Fig. S6. Metabolic pathways related to aromatic compounds degradation identified in LIgMet according to KEGG automatic annotation.

Fig. S7. Classification of the predicted proteins from LigMet metagenome according to the dbCAN database.

Fig. S8. Predicted auxiliary activity (AA) and carbohydrate esterase (CE) families from LigMet and draft genomes, based on the dbCAN database. AA and CE families are related to peroxidase activity and break down of lignin ester cross links, respectively.

Fig S9. Phylogenetic position of strain HW13 relative to the most closely related strains of the genus *Paenarthrobacter.* EzBioCloud webserver [5] was used to perform a similarity-based search of HW13 16S rRNA to retrieve the most closely related sequences. The resulting 16S rRNA sequences were aligned using MAFFT v7.299b software [6]. A phylogenetic tree was inferred using maximum likelihood method implemented in RAxML v8.2.0 [7], evolutionary distances were based on the GTRGAMMAI model, inferred as the best model by jModelTest2 [8]. Numbers at the nodes are percentages of bootstrap values obtained by repeating the analysis 1,000 times to generate a consensus tree. The type strains are marked with a superscript ‘T’. Accession numbers are shown in parentheses.

Fig S10. Phylogenetic relationships among feruloyl-CoA synthetase (upper) and Enoyl-CoA hydratase/aldolase**.** The phylogenetic tree was generated using amino acid sequences retrieved from NCBI

and Uniprot database. The sequences were aligned using MAFFT v7.299b software [6]. The phylogenetic tree was reconstructed using maximum likelihood method implemented in RAxML v8.2.0 [7], evolutionary distances were based on the GTRGAMMAI model, inferred as the best model by jModelTest2 [8]. The bootstrap values (1,000 replicate runs, shown as %) higher than 70 % are represented. Accession numbers are listed in parentheses. The FerA_B3 and FerB_B11 amino acid sequence retrieved from LigMet dataset is printed in bold.

References

1. Aslanidis C, Jong PJ De. Ligation-independent cloning of PCR products (LIC-PCR). Nucleic Acids Res. 1990;18:6069–74.

2. Rocha GJM, Martín C, da Silva VFN, Gómez EO, Gonçalves AR. Mass balance of pilot-scale pretreatment of sugarcane bagasse by steam explosion followed by alkaline delignification. Bioresour. Technol. Elsevier Ltd; 2012;111:447–52.

3. Suguiyama VF, Silva E a, Meirelles ST, Centeno DC, Braga MR. Leaf metabolite profile of the Brazilian resurrection plant Barbacenia purpurea Hook. (Velloziaceae) shows two time-dependent responses during desiccation and recovering. Front. Plant Sci. 2014;5:96.

4. Machida RJ, Knowlton N. PCR Primers for Metazoan Nuclear 18S and 28S Ribosomal DNA Sequences. PLoS One. 2012;7.

5. Yoon SH, Ha SM, Kwon S, Lim J, Kim Y, Seo H, et al. Introducing EzBioCloud: A taxonomically united database of 16S rRNA gene sequences and whole-genome assemblies. Int. J. Syst. Evol. Microbiol. 2017;67:1613–7.

6. Katoh K, Standley DM. MAFFT multiple sequence alignment software version 7: Improvements in performance and usability. Mol. Biol. Evol. 2013;30:772–80.

7. Stamatakis A. RAxML version 8: A tool for phylogenetic analysis and post-analysis of large phylogenies. Bioinformatics. 2014;30:1312–3.

8. Darriba D, Taboada GL, Doallo R, Posada D. JModelTest 2: More models, new heuristics and parallel computing. Nat. Methods 2012;9:772.

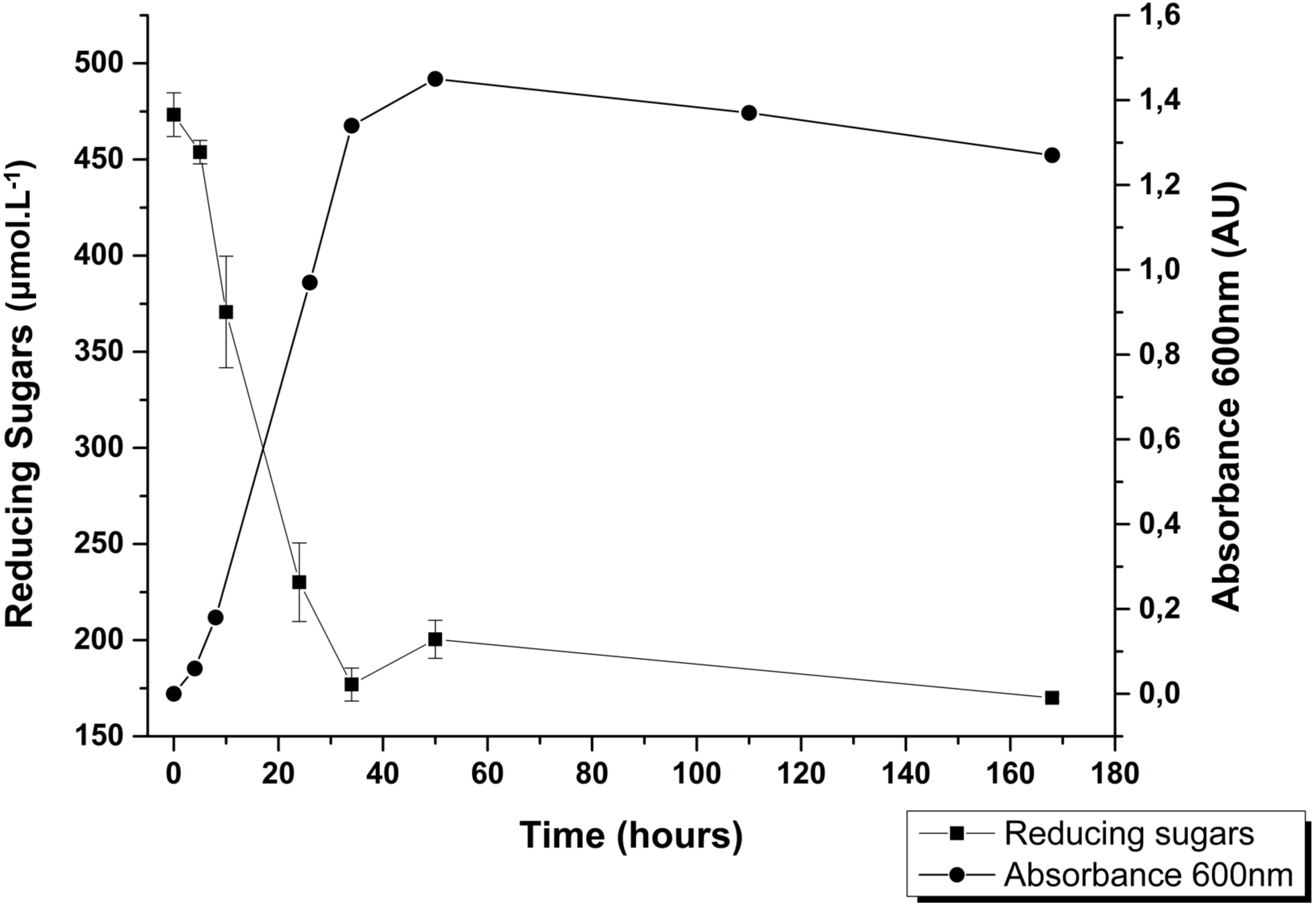

Reducing Sugars (µmol.L-1)
Absorbance 600nm (AU)
Time (hours)
500
450
400
350
300
250
200
150
1,6
1,4
1,2
1,0
0,8
0,6
0,4
0,2
0,0
0
20
40
60
80
100
120
140
160
180
Reducing sugars
Absorbance 600nm

Figure S1

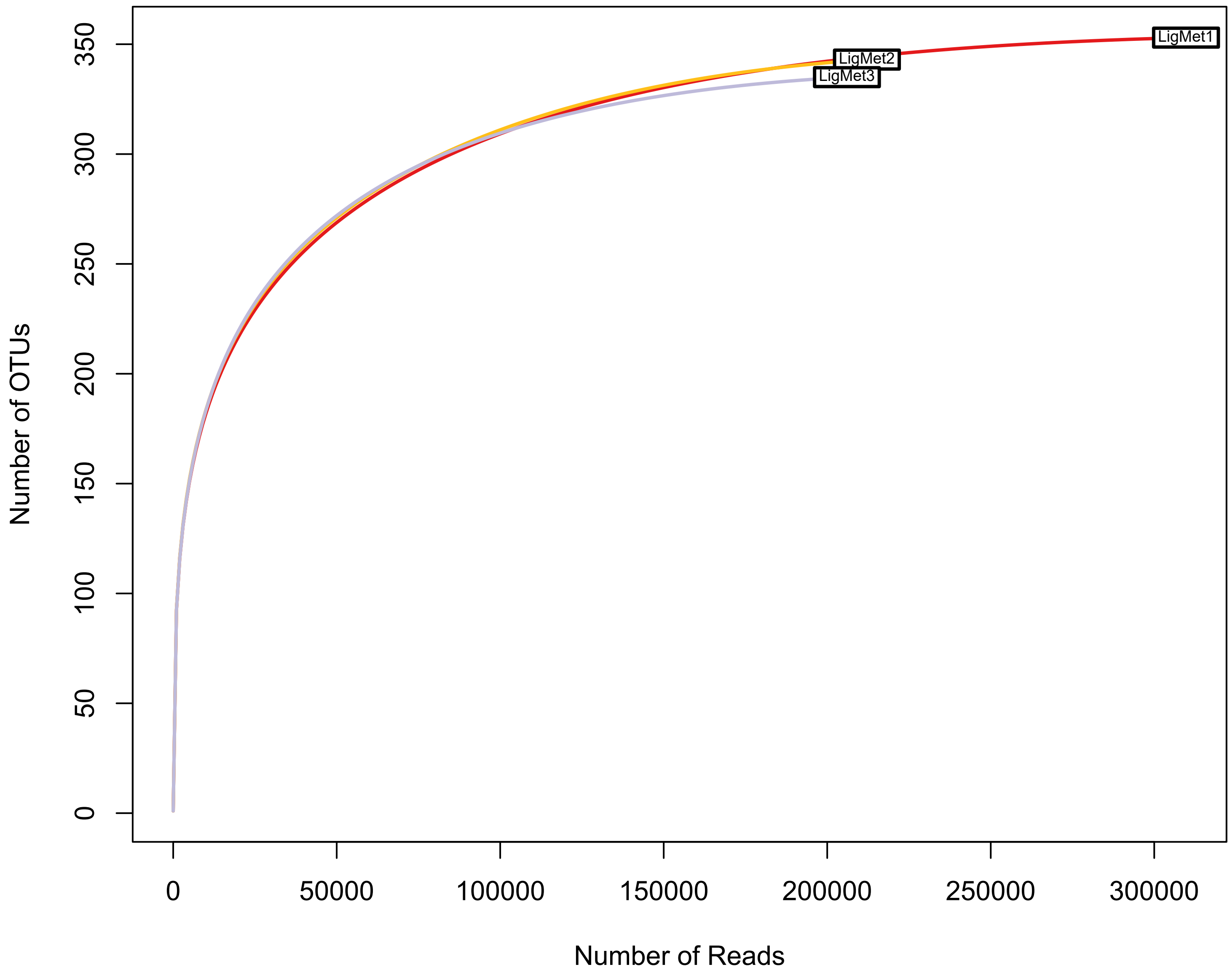
LigMet1
LigMet2
LigMet3
Number of OTUs
0
50
100
150
200
250
300
350
0
50000
100000
150000
200000
250000
300000
Number of Reads

Figure S2A

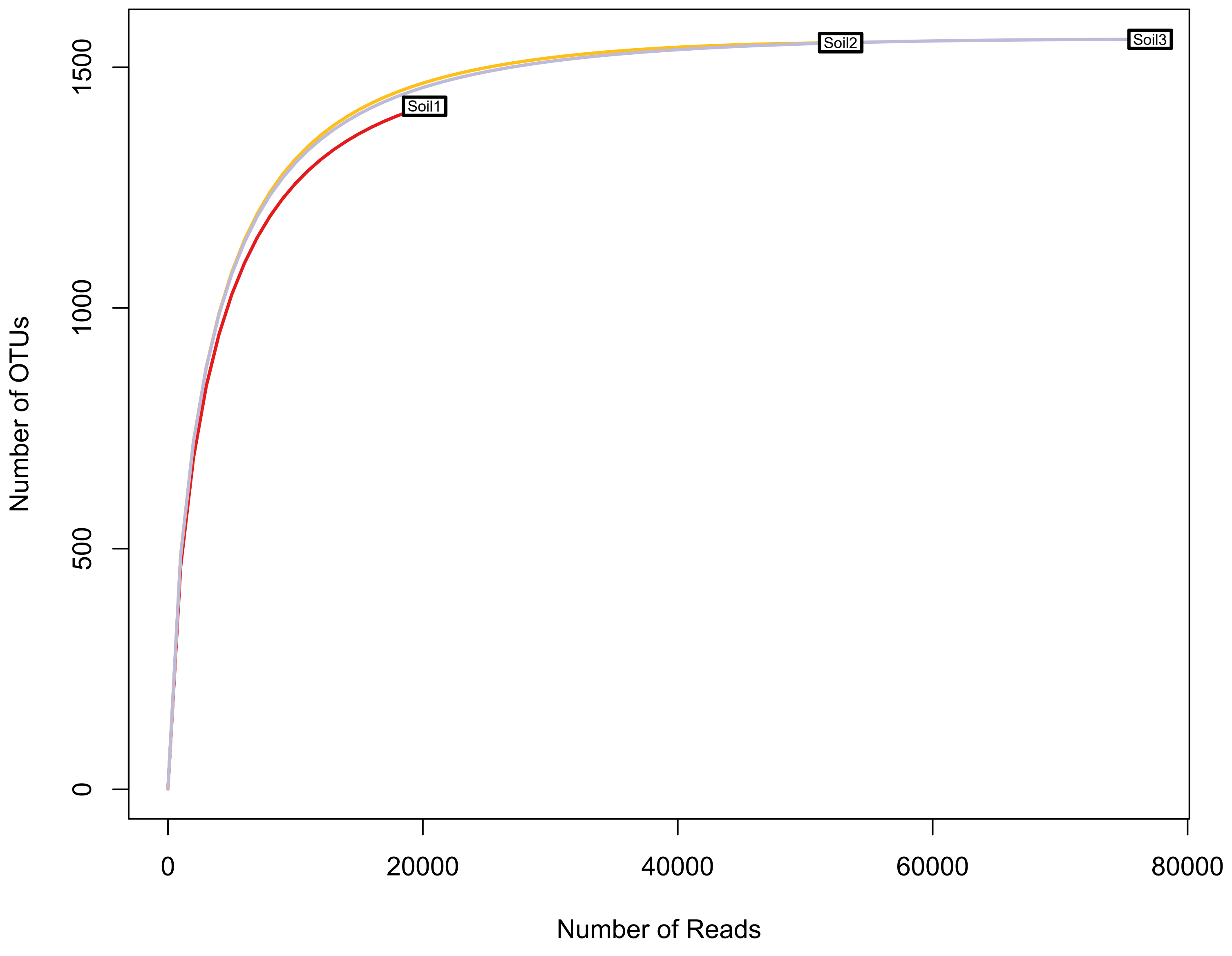
Soil1
Soil2
Soil3
0
500
1000
1500
Number of OTUs
0
20000
40000
60000
80000
Number of Reads

Figure S2B

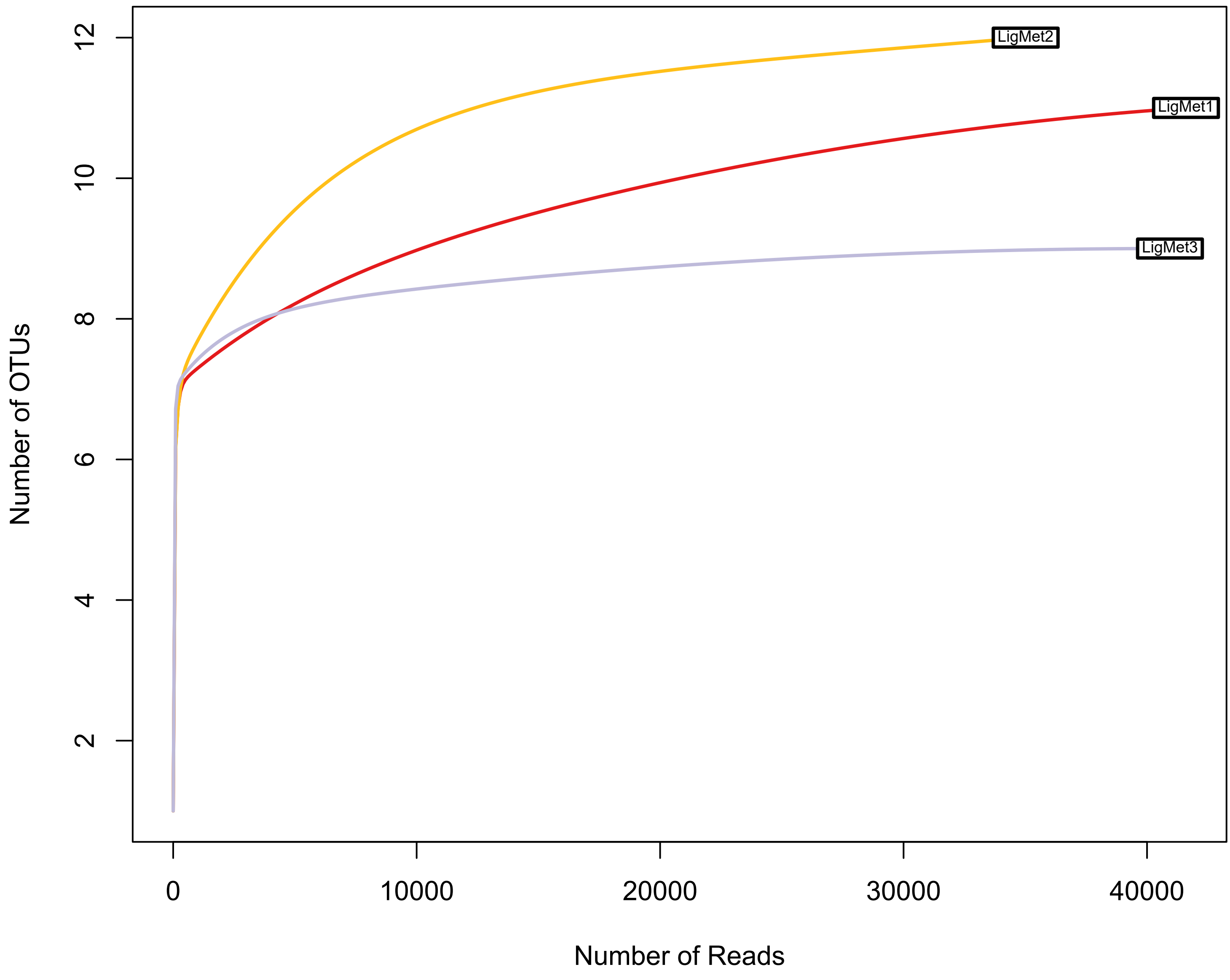
LigMet2
LigMet1
LigMet3
12
10
8
6
4
2
Number of OTUs
0
10000
20000
30000
40000
Number of Reads

Figure S3

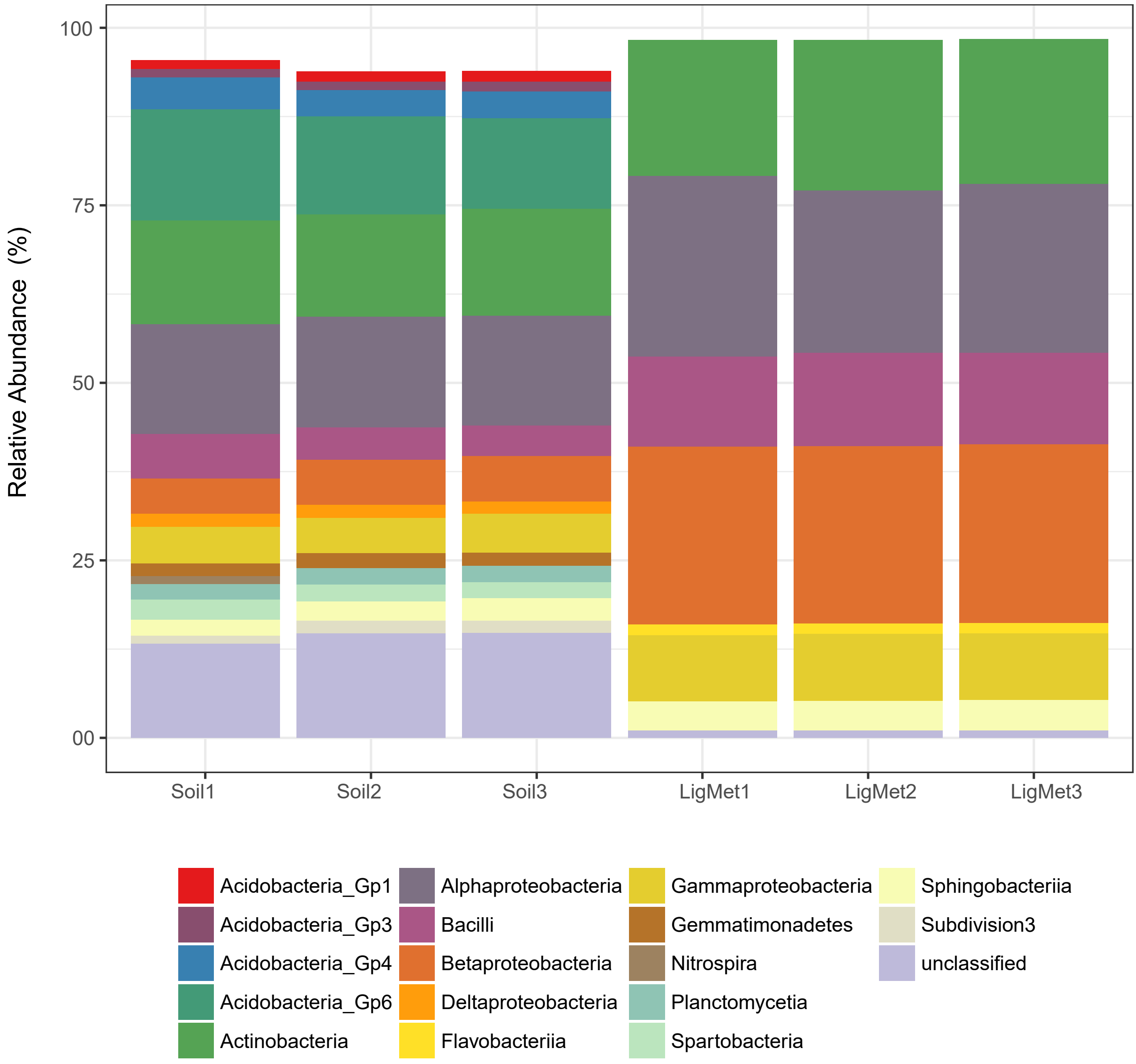

Relative Abundance (%)
100
75
50
25
00
Soil1
Soil2
Soil3
LigMet1
LigMet2
LigMet3
Acidobacteria_Gp1
Acidobacteria_Gp3
Acidobacteria_Gp4
Acidobacteria_Gp6
Actinobacteria
Alphaproteobacteria
Bacilli
Betaproteobacteria
Deltaproteobacteria
Flavobacteriia
Gammaproteobacteria
Gemmatimonadetes
Nitrospira
Planctomycetia
Spartobacteria
Sphingobacteriia
Subdivision3
unclassified

Figure S4

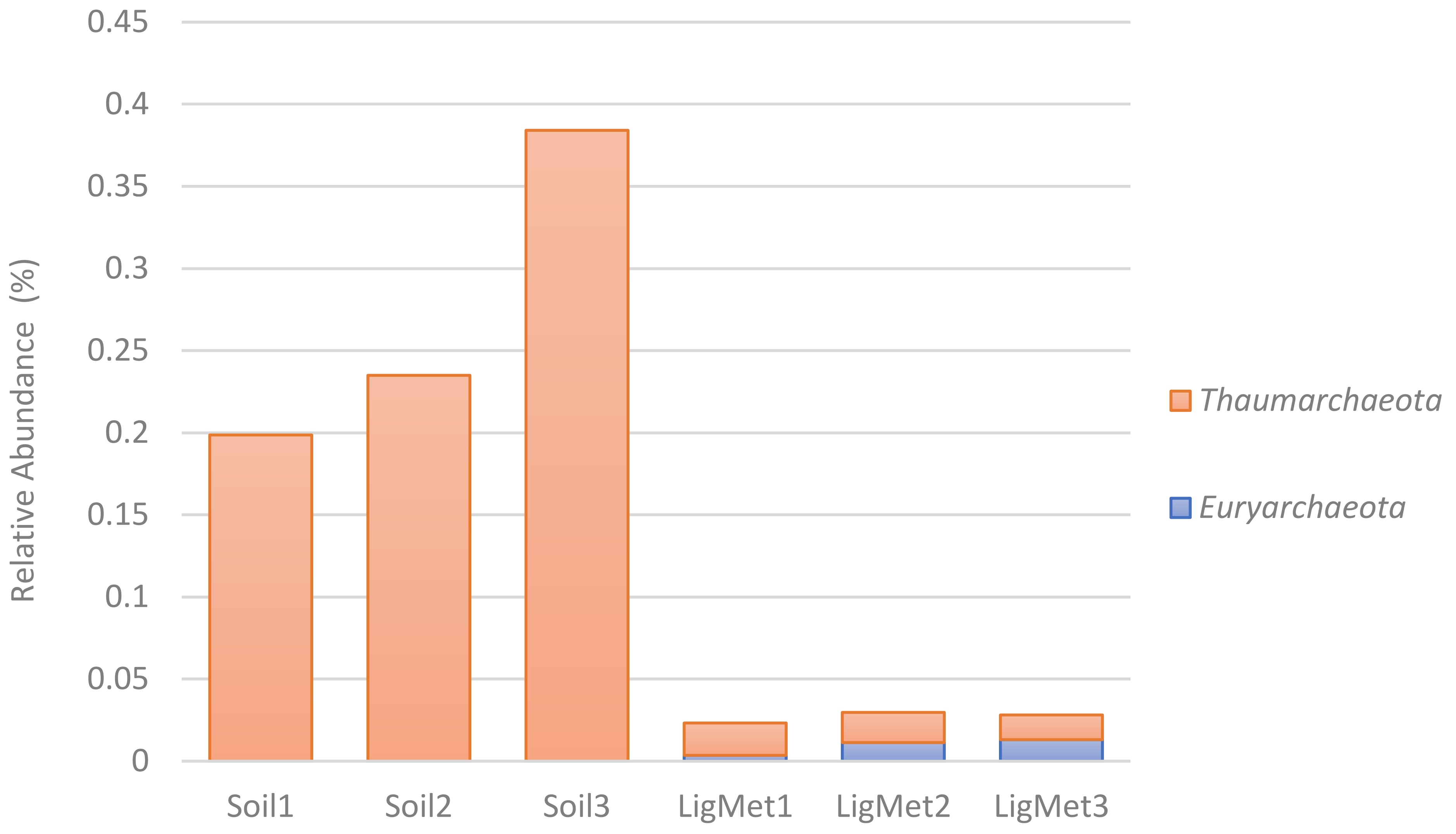

0.45
0.4
0.35
0.3
0.25
0.2
0.15
0.1
0.05
0
Relative Abundance (%)
Soil1
Soil2
Soil3
LigMet1
LigMet2
LigMet3
Thaumarchaeota
Euryarchaeota

Figure S5

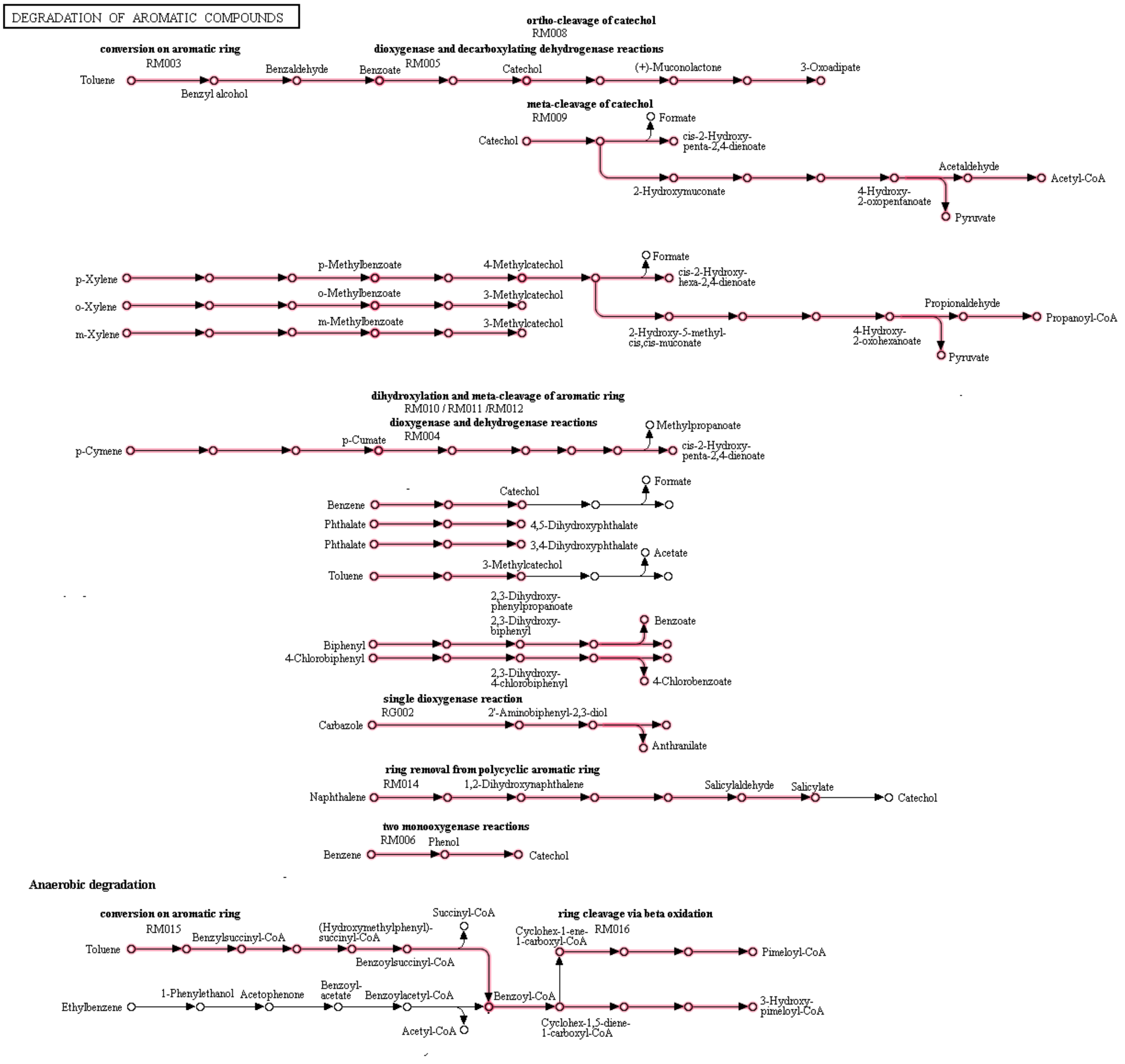
DEGRADATION OF AROMATIC COMPOUNDS
ortho-cleavage of catechol
RM008
conversion on aromatic ring
RM003
dioxygenase and decarboxylating dehydrogenase reactions
RM005
Toluene
Benzyl alcohol
Benzaldehyde
Benzoate
Catechol
(+)-Muconolactone
3-Oxoadipate
meta-cleavage of catechol
RM009
Formate
Catechol
cis-2-Hydroxy-penta-2,4-dienoate
2-Hydroxymuconate
Acetaldehyde
Acetyl-CoA
4-Hydroxy-2-oxopentanoate
Pyruvate
p-Xylene
o-Xylene
m-Xylene
p-Methylbenzoate
o-Methylbenzoate
m-Methylbenzoate
4-Methylcatechol
3-Methylcatechol
3-Methylcatechol
Formate
cis-2-Hydroxy-hexa-2,4-dienoate
Propionaldehyde
Propanoyl-CoA
2-Hydroxy-5-methyl-cis,cis-muconate
4-Hydroxy-2-oxohexanoate
Pyruvate
dihydroxylation and meta-cleavage of aromatic ring
RM010 / RM011 /RM012
dioxygenase and dehydrogenase reactions
RM004
Methylpropanoate
p-Cymene
p-Cumate
cis-2-Hydroxy-penta-2,4-dienoate
Formate
Benzene
Catechol
Phthalate
4,5-Dihydroxyphthalate
Phthalate
3,4-Dihydroxyphthalate
Acetate
Toluene
3-Methylcatechol
2,3-Dihydroxy-phenylpropanoate
2,3-Dihydroxy-biphenyl
Benzoate
Biphenyl
4-Chlorobiphenyl
2,3-Dihydroxy-4-chlorobiphenyl
4-Chlorobenzoate
single dioxygenase reaction
RG002
2'-Aminobiphenyl-2,3-diol
Carbazole
Anthranilate
ring removal from polycyclic aromatic ring
RM014
1,2-Dihydroxynaphthalene
Naphthalene
Salicylaldehyde
Salicylate
Catechol
two monooxygenase reactions
RM006
Phenol
Benzene
Catechol
Anaerobic degradation
conversion on aromatic ring
RM015
Succinyl-CoA
ring cleavage via beta oxidation
RM016
Toluene
Benzylsuccinyl-CoA
(Hydroxymethylphenyl)-succinyl-CoA
Benzoylsuccinyl-CoA
Cyclohex-1-ene-1-carboxyl-CoA
Pimeloyl-CoA
Ethylbenzene
1-Phenylethanol
Acetophenone
Benzoyl-acetate
Benzoylacetyl-CoA
Benzoyl-CoA
Acetyl-CoA
Cyclohex-1,5-diene-1-carboxyl-CoA
3-Hydroxy-pimeloyl-CoA

Figure S6

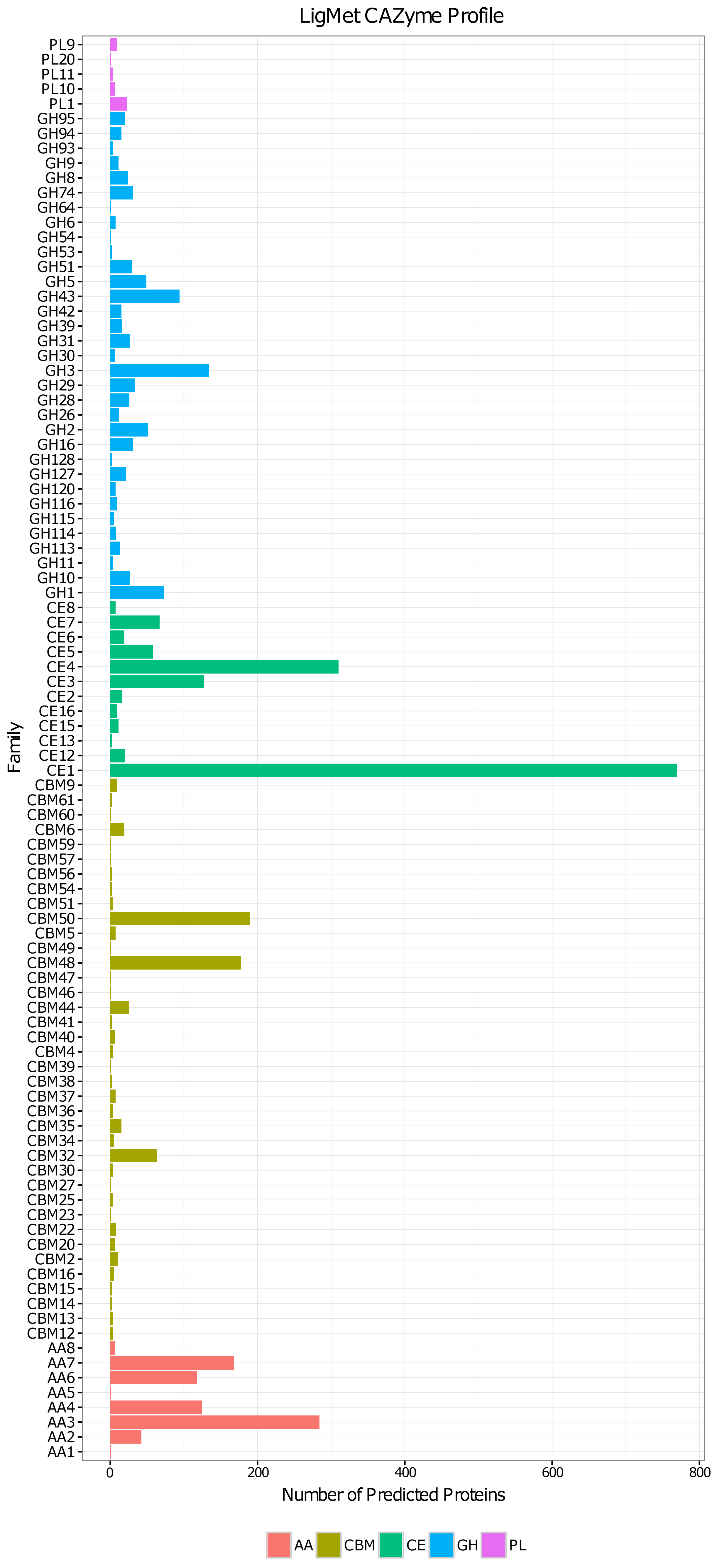

LigMet CAZyme Profile
PL9
PL20
PL11
PL10
PL1
GH95
GH94
GH93
GH9
GH8
GH74
GH64
GH6
GH54
GH53
GH51
GH5
GH43
GH42
GH39
GH31
GH30
GH3
GH29
GH28
GH26
GH2
GH16
GH128
GH127
GH120
GH116
GH115
GH114
GH113
GH11
GH10
GH1
CE8
CE7
CE6
CE5
CE4
CE3
CE2
CE16
CE15
CE13
CE12
CE1
CBM9
CBM61
CBM60
CBM6
CBM59
CBM57
CBM56
CBM54
CBM51
CBM50
CBM5
CBM49
CBM48
CBM47
CBM46
CBM44
CBM41
CBM40
CBM4
CBM39
CBM38
CBM37
CBM36
CBM35
CBM34
CBM32
CBM30
CBM27
CBM25
CBM23
CBM22
CBM20
CBM2
CBM16
CBM15
CBM14
CBM13
CBM12
AA8
AA7
AA6
AA5
AA4
AA3
AA2
AA1
Family
0
200
400
600
800
Number of Predicted Proteins
AA
CBM
CE
GH
PL

Figure S7

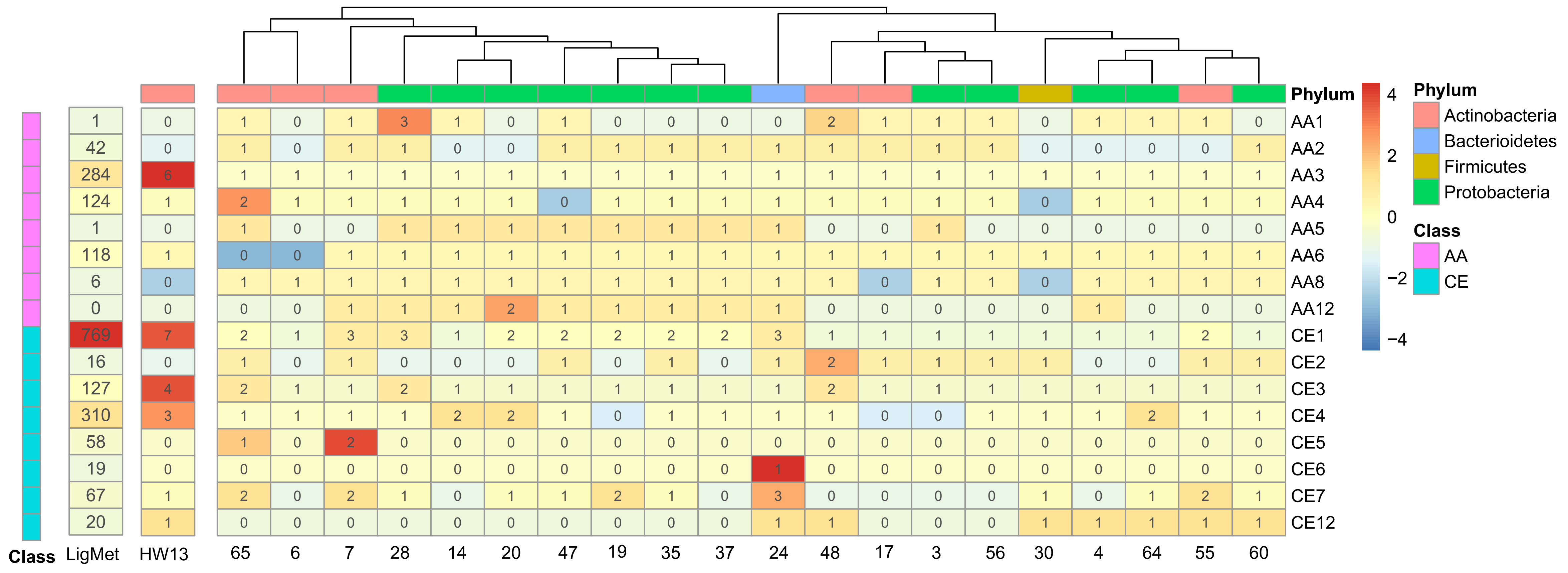
Phylum
Actinobacteria
Bacterioidetes
Firmicutes
Protobacteria
Class
AA
CE
4
2
0
−2
−4
Class LigMet HW13 65 6 7 28 14 20 47 19 35 37 24 48 17 3 56 30 4 64 55 60
AA1 1 0 1 0 1 3 1 0 1 0 0 0 0 2 1 1 1 0 1 1 1 0
AA2 42 0 1 0 1 1 0 0 1 1 1 1 1 1 1 1 1 0 0 0 0 1
AA3 284 6 1 1 1 1 1 1 1 1 1 1 1 1 1 1 1 1 1 1 1 1
AA4 124 1 2 1 1 1 1 1 0 1 1 1 1 1 1 1 1 0 1 1 1 1
AA5 1 0 1 0 0 1 1 1 1 1 1 1 1 0 0 1 0 0 0 0 0 0
AA6 118 1 0 0 1 1 1 1 1 1 1 1 1 1 1 1 1 1 1 1 1 1
AA8 6 0 1 1 1 1 1 1 1 1 1 1 1 1 0 1 1 0 1 1 1 1
AA12 0 0 0 0 1 1 1 2 1 1 1 1 1 0 0 0 0 0 1 0 0 0
CE1 769 7 2 1 3 3 1 2 2 2 2 2 3 1 1 1 1 1 1 1 2 1
CE2 16 0 1 0 1 0 0 0 1 0 1 0 1 2 1 1 1 1 0 0 1 1
CE3 127 4 2 1 1 2 1 1 1 1 1 1 1 2 1 1 1 1 1 1 1 1
CE4 310 3 1 1 1 1 2 2 1 0 1 1 1 1 0 0 1 1 1 2 1 1
CE5 58 0 1 0 2 0 0 0 0 0 0 0 0 0 0 0 0 0 0 0 0 0
CE6 19 0 0 0 0 0 0 0 0 0 0 0 1 0 0 0 0 0 0 0 0 0
CE7 67 1 2 0 2 1 0 1 1 2 1 0 3 0 0 0 0 1 0 1 2 1
CE12 20 1 0 0 0 0 0 0 0 0 0 0 1 1 0 0 0 1 1 1 1 1

Figure S8

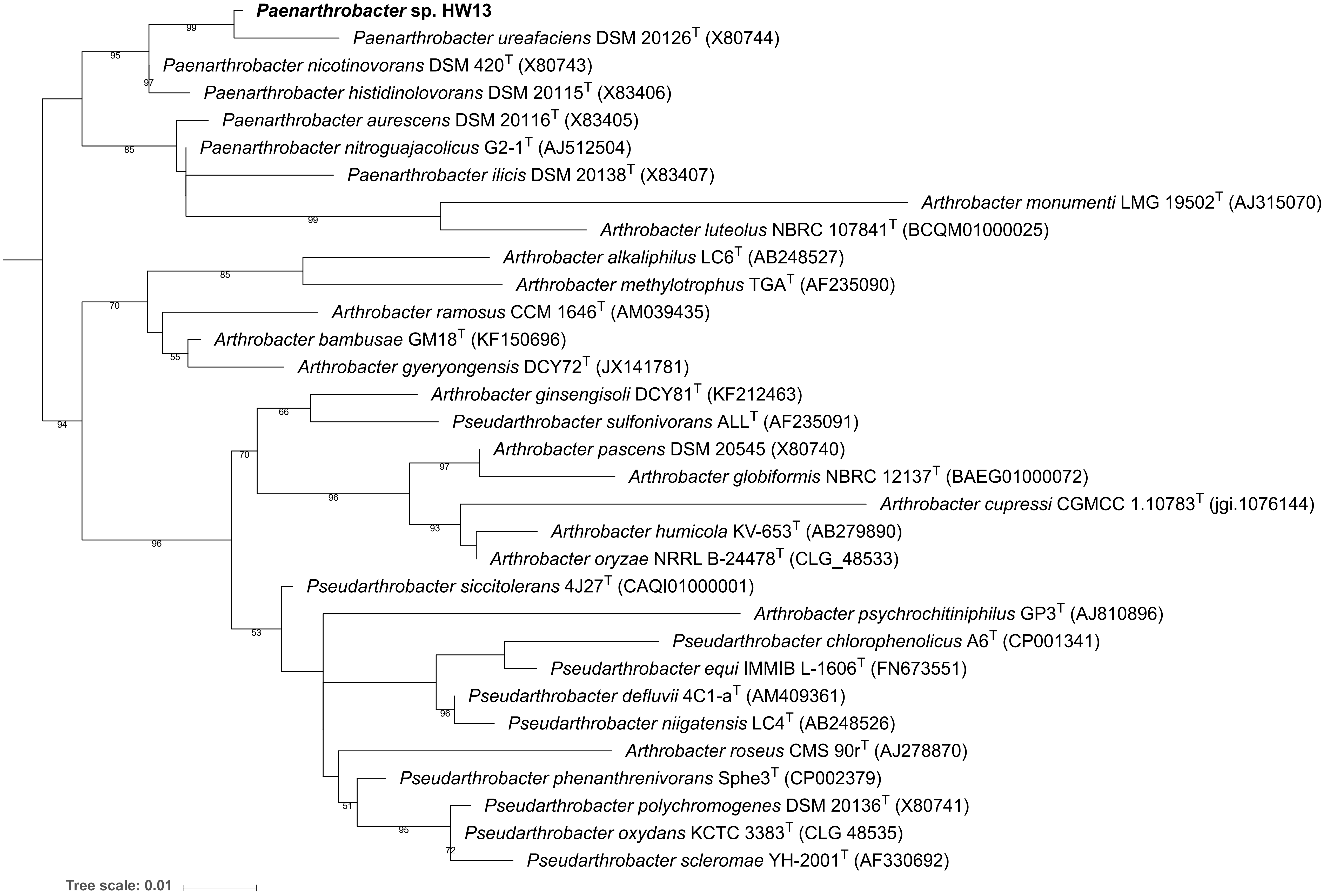
Paenarthrobacter sp. HW13
Paenarthrobacter ureafaciens DSM 20126T (X80744)
Paenarthrobacter nicotinovorans DSM 420T (X80743)
Paenarthrobacter histidinolovorans DSM 20115T (X83406)
Paenarthrobacter aurescens DSM 20116T (X83405)
Paenarthrobacter nitroguajacolicus G2-1T (AJ512504)
Paenarthrobacter ilicis DSM 20138T (X83407)
Arthrobacter monumenti LMG 19502T (AJ315070)
Arthrobacter luteolus NBRC 107841T (BCQM01000025)
Arthrobacter alkaliphilus LC6T (AB248527)
Arthrobacter methylotrophus TGAT (AF235090)
Arthrobacter ramosus CCM 1646T (AM039435)
Arthrobacter bambusae GM18T (KF150696)
Arthrobacter gyeryongensis DCY72T (JX141781)
Arthrobacter ginsengisoli DCY81T (KF212463)
Pseudarthrobacter sulfonivorans ALLT (AF235091)
Arthrobacter pascens DSM 20545 (X80740)
Arthrobacter globiformis NBRC 12137T (BAEG01000072)
Arthrobacter cupressi CGMCC 1.10783T (jgi.1076144)
Arthrobacter humicola KV-653T (AB279890)
Arthrobacter oryzae NRRL B-24478T (CLG_48533)
Pseudarthrobacter siccitolerans 4J27T (CAQI01000001)
Arthrobacter psychrochitiniphilus GP3T (AJ810896)
Pseudarthrobacter chlorophenolicus A6T (CP001341)
Pseudarthrobacter equi IMMIB L-1606T (FN673551)
Pseudarthrobacter defluvii 4C1-aT (AM409361)
Pseudarthrobacter niigatensis LC4T (AB248526)
Arthrobacter roseus CMS 90rT (AJ278870)
Pseudarthrobacter phenanthrenivorans Sphe3T (CP002379)
Pseudarthrobacter polychromogenes DSM 20136T (X80741)
Pseudarthrobacter oxydans KCTC 3383T (CLG 48535)
Pseudarthrobacter scleromae YH-2001T (AF330692)
99
95
97
85
99
85
70
55
66
70
97
96
93
96
53
96
51
95
72
Tree scale: 0.01

Figure S9

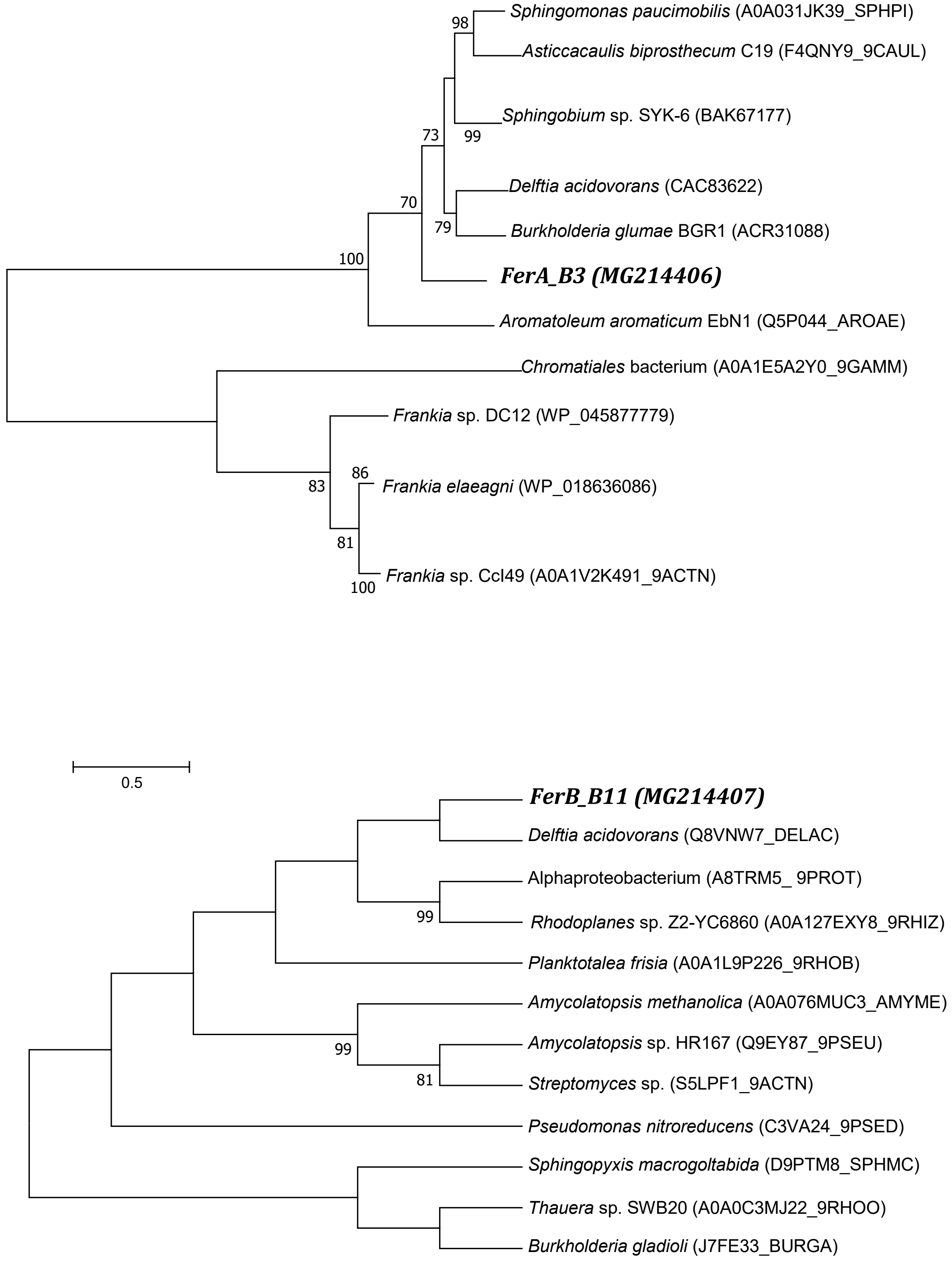

Sphingomonas paucimobilis (A0A031JK39_SPHPI)
Asticcacaulis biprosthecum C19 (F4QNY9_9CAUL)
Sphingobium sp. SYK-6 (BAK67177)
Delftia acidovorans (CAC83622)
Burkholderia glumae BGR1 (ACR31088)
FerA_B3 (MG214406)
Aromatoleum aromaticum EbN1 (Q5P044_AROAE)
Chromatiales bacterium (A0A1E5A2Y0_9GAMM)
Frankia sp. DC12 (WP_045877779)
Frankia elaeagni (WP_018636086)
Frankia sp. CcI49 (A0A1V2K491_9ACTN)
98
99
73
70
79
100
83
86
81
100
0.5
FerB_B11 (MG214407)
Delftia acidovorans (Q8VNW7_DELAC)
Alphaproteobacterium (A8TRM5_ 9PROT)
Rhodoplanes sp. Z2-YC6860 (A0A127EXY8_9RHIZ)
Planktotalea frisia (A0A1L9P226_9RHOB)
Amycolatopsis methanolica (A0A076MUC3_AMYME)
Amycolatopsis sp. HR167 (Q9EY87_9PSEU)
Streptomyces sp. (S5LPF1_9ACTN)
Pseudomonas nitroreducens (C3VA24_9PSED)
Sphingopyxis macrogoltabida (D9PTM8_SPHMC)
Thauera sp. SWB20 (A0A0C3MJ22_9RHOO)
Burkholderia gladioli (J7FE33_BURGA)
99
99
81

Figure S10
